# Proteomics of circulating extracellular vesicles reveals diverse clinical presentations of COVID-19 but fails to identify viral peptides

**DOI:** 10.3389/fcimb.2024.1442743

**Published:** 2024-11-06

**Authors:** Melisa Gualdrón-López, Alberto Ayllon-Hermida, Núria Cortes-Serra, Patricia Resa-Infante, Joan Josep Bech-Serra, Iris Aparici-Herraiz, Marc Nicolau-Fernandez, Itziar Erkizia, Lucia Gutierrez-Chamorro, Silvia Marfil, Edwards Pradenas, Carlos Ávila Nieto, Bernat Cucurull, Sergio Montaner-Tarbés, Magdalena Muelas, Ruth Sotil, Ester Ballana, Victor Urrea, Lorenzo Fraile, Maria Montoya, Julia Vergara, Joaquim Segales, Jorge Carrillo, Nuria Izquierdo-Useros, Julià Blanco, Carmen Fernandez-Becerra, Carolina de La Torre, Maria-Jesus Pinazo, Javier Martinez-Picado, Hernando A. del Portillo

**Affiliations:** ^1^ ISGlobal, Hospital Clínic - Universitat de Barcelona, Barcelona, Spain; ^2^ Germans Trias I Pujol Research Institute (IGTP), Badalona, Spain; ^3^ IrsiCaixa, Badalona, Spain; ^4^ CIBERINFEC, Instituto de Salud Carlos III, Madrid, Spain; ^5^ University of Vic–Central University of Catalonia (UVic-UCC), Vic, Spain; ^6^ Josep Carreras Leukemia Research Institute, Badalona, Spain; ^7^ Innovex Therapeutics S.L., Badalona, Spain; ^8^ International Health Service, Hospital Clínic, Barcelona, Spain; ^9^ Agro tecnio Center, Department of Animal Science, University of Lleida, Lleida, Spain; ^10^ Centro de Investigaciones Biológicas Margarita Salas (CIB-CSIC), Madrid, Spain; ^11^ Unitat Mixta d’Investigació Institute of Agrifood Research and Technology UAB (IRTA-UAB) en Sanitat Animal, Centre de Recerca en Sanitat Animal (CReSA), Bellaterra, Spain; ^12^ Institute of Agrifood Research and Technology (IRTA) Programa de Sanitat Animal, Centre de Recerca en Sanitat Animal (CReSA), Bellaterra, Spain; ^13^ Departament de Sanitat i Anatomia Animals, Facultat de Veterinària, Bellaterra, Spain; ^14^ Catalan Institution for Research and Advanced Studies (ICREA), Barcelona, Spain

**Keywords:** COVID-19 patients, SARS-CoV-2, antibody response, extracellular vesicles, immunocapture (CD9), ganglioside-capture (CD169/Siglec-1), size-exclusion chromatography (SEC), proteomics profiling

## Abstract

Extracellular vesicles (EVs) released by virus-infected cells have the potential to encapsulate viral peptides, a characteristic that could facilitate vaccine development. Furthermore, plasma-derived EVs may elucidate pathological changes occurring in distal tissues during viral infections. We hypothesized that molecular characterization of EVs isolated from COVID-19 patients would reveal peptides suitable for vaccine development. Blood samples were collected from three cohorts: severe COVID-19 patients (G1), mild/asymptomatic cases (G2), and SARS-CoV-2-negative healthcare workers (G3). Samples were obtained at two time points: during the initial phase of the pandemic in early 2020 (m0) and eight months later (m8). Clinical data analysis revealed elevated inflammatory markers in G1. Notably, non-vaccinated individuals in G1 exhibited increased levels of neutralizing antibodies at m8, suggesting prolonged exposure to viral antigens. Proteomic profiling of EVs was performed using three distinct methods: immunocapture (targeting CD9), ganglioside-capture (utilizing Siglec-1) and size-exclusion chromatography (SEC). Contrary to our hypothesis, this analysis failed to identify viral peptides. These findings were subsequently validated through Western blot analysis targeting the RBD of the SARS-CoV-2 Spike protein’s and comparative studies using samples from experimentally infected Syrian hamsters. Furthermore, analysis of the EV cargo revealed a diverse molecular profile, including components involved in the regulation of viral replication, systemic inflammation, antigen presentation, and stress responses. These findings underscore the potential significance of EVs in the pathogenesis and progression of COVID-19.

## Introduction

Coronavirus Disease (COVID-19) is an acute infectious condition caused by the SARS-CoV-2 virus, affecting more than 676 million people worldwide and resulting in over 6.8 million deaths (https://coronavirus.jhu.edu/map.html) (Zhu et al., 2020). COVID-19 patients can manifest a wide spectrum of symptoms, ranging from mild to severe. Some severely affected individuals rapidly progress to conditions such as pneumonia, systemic life-threatening disorders, and in some cases, succumb to the disease (Chams et al., 2020; Huang et al., 2020). Severe COVID-19 primarily arises from an uncontrolled immune and inflammatory response triggered by viral replication in the lungs (Boechat et al., 2021; Merad et al., 2022), leading to the development of severe respiratory illness reminiscent of the severe acute respiratory syndrome (Huang et al., 2020; Wiersinga et al., 2020).

Extracellular vesicles (EVs) are lipid bilayer-enclosed, cell-derived nanoparticles, typically ranging in size from 30 nm to 1 μm in diameter (Yanez-Mo et al., 2015). They are secreted by all cellular organisms and play crucial roles in facilitating intercellular communication (Raposo and Stahl, 2023). EVs follow a similar assembly pathway to some viruses, as both are generated within multivesicular bodies. This process leads to the release of viral proteins and nucleic acids into the extracellular space, potentially triggering immune responses (Purvinsh et al., 2021). Notably, previous research has discovered immunogenic viral peptides in circulating EVs from a single-stranded RNA virus responsible for porcine respiratory distress (Montaner-Tarbes et al., 2016, 2018), and increasing evidence supports the use of EVs in immunotherapy and vaccination, including EV-vaccines against SARS-CoV-2 (Sabanovic et al., 2021; Buzas, 2023; Kalluri, 2024). Building upon this knowledge, we hypothesized that a comprehensive analysis of circulating EVs in COVID-19 patients with diverse clinical outcomes could reveal immunogenic peptides from SARS-CoV-2. These peptides may hold promise for vaccine development and biomarker discovery in the fight against COVID-19.

Here, we report that SARS-CoV-2 viral peptides were not detectable in EVs isolated through three distinct and complementary techniques: immunocapture (CD9), ganglioside-capture (Siglec-1) and size-exclusion chromatography (SEC). The absence of SARS-CoV-2 viral peptides in EVs was further validated through Western blot analysis of CD9/CD63/CD81-immunocaptured EVs, and proteomic analysis of EVs isolated from Syrian hamsters experimentally infected with SARS-CoV-2. Importantly, comprehensive characterization of the human EV cargo revealed a diverse molecular profile, including components involved in the regulation of viral replication, systemic inflammation, antigen presentation, and cellular stress responses. These findings highlight the potential role of EVs as mediators of host-pathogen interactions and disease progression in COVID-19.

## Materials and methods

### Subject details and experimental model

The study protocol was approved by the Ethics Committee of the Hospital Clinic of Barcelona (HCB/2020/0446). Individual written informed consent was obtained from all study participants before the collection of samples, after receiving oral and written complete information about study objectives and procedures.

Fifty-two participants were enrolled in the study and stratified in 3 different groups. A total of 15 individuals were included in group 1 (G1; COVID-19 patients with severe symptoms), 26 were included in group 2 (G2; COVID-19 patients with mild symptoms or asymptomatic), and 13 in group 3 (G3, uninfected healthcare workers at Hospital Clinic of Barcelona who performed their professional activities following the established protective measures for SARS-CoV-2). Individuals from G1 were hospitalized at conventional hospitalization rooms and at the severe intensive care unit (ICU), while individuals from G2 had an ambulatory management, with no need of hospitalization nor specific treatment.

### Hamster model

Animal experiments were approved by the Institutional Animal Welfare Committee of the Institut de Recerca i Tecnologia Agroalimentàries (CEEA-IRTA, registration number CEEA 188/2020) and by the Ethical Commission of Animal Experimentation of the Autonomous Government of Catalonia (registration number FUE-2020-01589810) and conducted by certified staff. Experiments with SARS-CoV-2 were performed at the Biosafety Level-3 (BSL-3) facilities of the Biocontainment Unit of IRTA-CReSA (Barcelona, Spain).

### Cell lines

HEK293T cells (ATCC repository) and HEK293T cells overexpressing WT human ACE-2 (Integral Molecular, USA) used as target in pseudovirus-based neutralization assay were maintained in T75 flasks with Dulbecco′s Modified Eagle′s Medium (DMEM) supplemented with 10% FBS and 1 µg/ml of Puromycin (Thermo Fisher Scientific, USA).

Expi293F cells (Thermo Fisher Scientific) are a HEK293 cell derivative adapted for suspension culture that were used for SARS-CoV-2 pseudovirus production. Cells were maintained under continuous shaking in Erlenmeyer flasks following manufacturer’s guidelines.

Vero E6 cells (ATCC CRL1586) were cultured in Dulbecco’s modified Eagle medium (Invitrogen) supplemented with 10% fetal bovine serum (FBS; Invitrogen), 100 U/mL penicillin, 100 μg/mL streptomycin (all from Invitrogen).

### Sample collection and procedures

At recruitment visits (infection peak for individuals of G1 and G2 = m0), a detailed evaluation of previous medical history, allergies, current treatment, symptoms and clinical signs by physical examination was made to all participants and registered in a CRF built *ad hoc*. Also, a nasopharyngeal swab was collected to conduct RT-PCR to detect SARS-CoV-2. For general laboratory tests, one sample of approximately 8.5 ml of blood was collected in rapid serum tubes (RST) from all participants. For serum purification, another 8.5 ml aliquot collected in RTS tubes was separated by centrifugation at 1600 xg for 10 min and samples aliquoted and stored at -80°C. In addition, one more sample of 10 ml of peripheral blood was withdrawn in citrate tubes and centrifuged at 700 xg for 15 min to separate the plasma from the cell pellet. Plasma was transferred to a new tube and centrifuged twice at 2000 xg for 10 min at 4°C to eliminate any cell debris. Plasma samples were aliquoted and stored at -80°C for later PCR analysis and purification of EVs. Patients willing to participate in the follow-up study were recruited eight months (m8) after the initial infection and submitted to a similar clinical evaluation. Collection of new samples in RST tubes and in citrate tubes was performed as described.

### SARS-CoV-2 PCR detection and viral load quantification in serum samples

All plasma samples used in this study were negative for SARS-CoV-2 as determined by Real-Time RT-PCR. RNA extraction was performed using Viral RNA/Pathogen Nucleic Acid Isolation kit (Cat number: A42352, Thermo Fisher), optimized for a KingFisher instrument (Thermo Fisher), following manufacturer’s instructions. PCR amplification was based on the 2019-Novel Coronavirus Real-Time RT-PCR Diagnostic Panel guidelines and protocol developed by the American Center for Disease control and Prevention time (https://www.fda.gov/media/134922/download). Briefly, a 20 μl PCR reaction was set up containing 5 μl of RNA, 1.5 μl of N2 or RNAseP primers and probe (2019-nCov CDC EUA Kit, Cat number: 10006770, Integrated DNA Technologies) and 10 μl of GoTaq 1-Step RT-qPCR (Promega). Thermal cycling was performed at 50°C for 15 min for reverse transcription, followed by 95°C for 2 min and then 45 cycles of 95°C for 10 sec, 56°C for 15 sec and 72°C for 30 sec in the Applied Biosystems 7500 or QuantStudio5 Real-Time PCR instruments (ThermoFisher). For absolute quantification, a standard curve was built using 1/5 serial dilutions of a SARS-CoV-2 plasmid (2019-nCoV_N_Positive Control, Cat number: 10006625, 200 copies/μl, Integrated DNA Technologies) and run in parallel in all PCR determinations. Viral load of each sample was determined in triplicate and mean viral load (in copies/ml) was extrapolated from the standard curve and corrected by the corresponding dilution factor. RNAseP gene amplification was performed in duplicate for each sample as amplification control.

### Determination of anti-SARS-CoV-2 antibodies by enzyme linked immunosorbent assay

The presence of anti-SARS-CoV-2 antibodies in plasma samples was evaluated using an in-house developed sandwich-ELISA. Nunc MaxiSorp ELISA plates were coated overnight at 4°C with 2g/ml of capture antibody (anti-6xHis antibody, clone HIS.H8; ThermoFisher Scientific) in PBS. PBS supplemented with 1% of bovine serum albumin (BSA, Miltenyi biotech) was used as a blocking buffer. Plates were incubated with blocking buffer for two hours at room temperature. Then, the SARS-CoV-2 Spike (S1+S2) or nucleocapside protein (NP) (both from Sino Biologicals) were added at 1g/ml in blocking buffer and incubated overnight at 4°C. Each plasma sample was evaluated in duplicated at dilution ranging from 1/200 - 1/1000 for each antigen. Diluted samples in blocking buffer were incubated at room temperature for 1h. Antigen free wells were also assayed in parallel for each sample in the same plate to evaluate samples background. Serial dilutions of a positive plasma sample were used as standard. A pool of 10 SARS-CoV-2 negative plasma samples, collected before June 2019, were included as negative control. HRP conjugated (Fab)2 Goat anti-human IgG (Fc specific) (1/20000), Goat anti-human IgM (1/10000), and Goat anti-human IgA (alpha chain specific) (1/10000) (all from Jackson Immunoresearch) were used as secondary antibodies and incubated for 30 minutes at room temperature. After washing, plates were revealed using o-Phenylenediamine dihydrochloride (OPD) (Sigma Aldrich) and the enzymatic reaction was stopped with 4N of H_2_SO_4_ (Sigma Aldrich). The signal was analyzed as the optical density (OD) at 492 nm with noise correction at 620 nm. The specific signal for each antigen was calculated after subtracting the background signal obtained for each sample in antigen-free wells. Standard curves were fitted to a 4-parameter logistic curve using the Prism 8.4.3 (GraphPad Software). Data are shown as arbitrary units (AU).

### Pseudovirus generation and neutralization assay

The plasmid pNL4-3.Luc.R-.E was obtained from BEI Resources (https://www.beiresources.org/). The plasmid SARS-CoV-2.SctΔ19 was generated (GeneArt) from the full protein sequence of the original WH1 SARS-CoV-2 spike (Genbank MN908947.3) with a deletion of the last 19 amino acids in C-terminal (Ou et al., 2020). The sequence was human-codon optimized and inserted into pcDNA3.1(+). Expi293F cells were transfected using ExpiFectamine293 Reagent (Thermo Fisher Scientific) with pNL4-3.Luc.R-.E- and SARS-CoV-2.SctΔ19 at an 8:1 ratio, respectively. Control pseudoviruses were obtained by replacing the S protein expression plasmid with a VSV-G protein expression plasmid. Supernatants were harvested 48h after transfection, filtered at 0.45 µm, frozen, and titrated on HEK293T cells overexpressing WT human ACE-2 (Integral Molecular, USA).

Neutralization assays were performed in duplicate. Briefly, in Nunc 96-well cell culture plates (Thermo Fisher Scientific), 200 TCID_50_ of pseudovirus were preincubated with three-fold serial dilutions (1/60–1/14,580) of heat-inactivated serum samples for 1h at 37°C. Then, 2x10^4^ HEK293T/hACE2 cells treated with DEAE-Dextran (Sigma-Aldrich) were added. Luciferase activity was read after 48 h using the EnSight Multimode Plate Reader and BriteLite Plus Luciferase reagent (PerkinElmer, USA). The values were normalized, and the ID_50_ (reciprocal dilution inhibiting 50% of the infection) was calculated by plotting and fitting all duplicate neutralization values and the log of plasma dilution to a 4-parameters equation in Prism 9.0.2 (GraphPad Software, USA). This neutralization assay has been previously validated in a large subset of samples and negative controls with a replicative viral inhibition assay (Mehrdad et al., 2021).

### SARS-CoV2 hamster *in vivo* model

Twelve Golden Syrian hamsters (Charles River, 5-6-week-old male and female) were inoculated by intranasal instillation with 10^5.8^ TCID_50_ of the SARS-CoV-2 Cat01 (D614G variant) isolate (CoV-19/Spain/CT-2020030095/2020; GISAID ID EPI_ISL_510689) per animal (100 μl/individual, 50 μl for each nostril), as previously reported (Brustolin et al., 2021). Briefly, four animals were sacrificed on days 2, 4 and 7 post-inoculation. Effective infection by SARS-CoV-2 was confirmed by genomic and subgenomic RNA RT-PCR, virus isolation and immunohistochemistry in the lungs, nasal turbinates, and trachea per each time-point. Three pools of 500 µl of serum were thermally inactivated 1h at 56°C before transferring from the BSL-3 for isolation of EVs. Of note, the effect of thermal inactivation for 1h at 56°C was previously assessed in plasma from BalbC mice. The recovery of EV in SEC was evaluated in parallel in treated and untreated plasma by using anti-CD5L Abs, with no differences in the efficiency of EV recovery.

### SARS-CoV-2 infection of Vero cells and lysate obtention for western blotting

4x10^6^ Vero cells were infected using a Multiplicity of Infection (MOI) of 0.02. After 48h, cells were washed with PBS and lysed with 1.5 ml of lysis buffer (2% SDS, 50mM Tris/HCl pH 7.6). As control, uninfected Vero cells were cultured under the same conditions and lysed after 48h with the same buffer. Both lysed cells were treated for 10 min at 95°C to be used later as control in Western Blotting.

### CD9 Direct immune-affinity capture of EVs from plasma

A total of 23 patient samples were used for CD9-immunocapture (8 G1, 10 G2, 5 G3) (**Supplementary Table 1**). Samples were chosen randomly and based on the volume available. One ml aliquots of human plasma samples were thawed on ice, immediately diluted 1:10 in cold PBS and ultracentrifuged at 120,000 xg for 4h in a TH-641 Swinging Bucket Rotor. Resulting pellets (p120 fraction) were resuspended in cold PBS and protein concentration determined by BCA (Biorad). 2000µg of total protein from p120 fractions were used as starting material for EVs immunocapture using CD9 Dynabeads™ (Thermo Fisher) following manufacturer’s instructions with slight modifications. Briefly, p120s fractions were incubated with 80 µl of CD9 Dynabeads™ (total volume of 400 µl) at 4°C with nutation and rotation for 16h. After incubation, flow-through was collected and stored. EVs-CD9 Dynabeads immunocomplexes were then washed twice with 1 ml and 0.5 ml ice-cold PBS, respectively. Immediately after the last wash, half of the sample (250 µl) was processed for proteomic analysis and half (250 µl) for western blotting.

### mS1 capture of EVs from plasma

12 patient samples (7 G1, 5 G3) were used for ms-1 capture (**Supplementary Table 1**). Samples were chosen randomly and based on the volume available. The plasmid pC-mS1-Fc was described before and encodes for a ms1 recombinant protein that consists of the V-set domain and three Ig-like domains of Siglec-1/CD169 cell receptor fused to the human IgG Fc domain (de Gassart et al., 2003). HEK293T cells were transfected using LipoD293 (Ver. II) Transfection Reagent (SignaGen Ref: SL100668) with 15 μg of that plasmid for 10^7^ cells in T75 flasks. Secreted mS1 protein was harvested 72h post-transfection, filtered at 0.22 µm and immediately frozen in 20% glycerol.

mS1-beads were prepared in a two-step procedure. First, 2.4·10^8^ of Aldehyde/Sulfate Latex beads (Thermo #A37304) were incubated with 0.32mg of recombinant protein G (Sigma-Aldrich # P4689) for 15 min in agitation at 20°C. Then, beads were saturated in 1% BSA. After two washing steps with cold PBS, beads were incubated with mS1 protein in saturating conditions for 30 min at room temperature in agitation. Then, beads were centrifuged for 3 min at 15,000 xg and washed three times with cold PBS.

For capturing, 1 ml aliquots of human plasma samples were thawed on ice, immediately diluted 1:10 in cold PBS and ultracentrifuged at 120,000 xg for 4h in a TH-641 Swinging Bucket Rotor. Resulting pellets (p120 fraction) were resuspended in cold PBS and protein concentration determined by BCA (Biorad). From P120 fractions described above, 1mg of total protein was incubated with 1.6·10^7^ mS1-B (total volume of 300 µl) at 20°C with agitation for 1h. Then, beads were centrifuged for 3 min at 15,000 xg. Flow-through was collected and stored. EVs-mS1-B complexes were then washed with 0.5 ml and 0.3 ml of ice-cold PBS. Immediately after the last wash, half of the sample (150 µl) was processed for proteomic analysis as described below.

### Size exclusion chromatography of plasma

Twelve patient samples were used for SEC EVs purification (4 G1, 6 G2 and 2 G3) (**Supplementary Table 1**). Samples were chosen randomly and based on the volume available. Briefly, one ml of human plasma from each sample, all previously stored at -80°C, were loaded onto 10 ml Izon qv Original (70nm) columns (one per group) following manufacturers´ instructions. Briefly, columns were washed with two volumes of sterile PBS 1X before loading plasma samples into the columns. Thirteen fractions of 500 ul each were collected using the qEV automatic fraction collector (Izon). After running each sample, columns were flushed with 1.5 column volumes of sterile PBS 1X and the next sample processed as recommended by the Izon qEV Original technical notes. A maximum of five samples from a group were passed through the same individual column.

For EVs isolation from SARS-CoV-2 infected hamsters, three different plasma samples were evaluated (sera pool from 4 animals at 2-, 4- and 7-days post-infection with SARS-CoV-2 and heat inactivated at 56°C/1h). SEC was employed for EVs isolation using individual 10 ml Izon qv Original (70 nm) columns were used for each sample following the procedure described above for human plasma samples.

### CD9/CD63/CD81 Direct Immune-affinity capture (DIC) of EVs from plasma

One hundred microliters aliquots of human plasma samples were thawed on ice, immediately used as starting material for EVs immunocapture using EasySep™ Pan-Human Extracellular vesicles positive selection kit (Stem Cell) following the manufacturer’s instructions.

### Nanoparticle tracking analysis

SEC fractions from patients in G1, G2 and G3 were analyzed by Nanoparticle Tracking analysis using NanoSight LM10- 12 instrument (Malvern Instruments Ltd, Malvern, UK) as previously reported (de Menezes-Neto et al., 2015) using the NTA software (version 3.2). Also, p120 fractions of plasma used for CD9 capture and mS1 capture were analyzed by NTA as described above.

### Western blotting

CD9*^+^* EVs-Dynabeads or CD9^+^/CD63^+^/CD81^+^immunocomplexes were resuspended in 20 µl of Lysis buffer (20 mM Tris-HCl pH 7.5, 150 mM NaCl, 1 mM Na_2_EDTA, 1 mM EGTA, 1% Triton X-100), sonicated and incubated for 30 minutes on ice. Lysed EVs protein concentration was determined by BCA (BioRad). EVs Lysates were then mixed with NuPAGE LDS Sample Buffer (4X) (Invitrogen, NP0007) in the presence or absence of 1mM DTT. For CD9 and CD81 immunoblots of CD9^+^EVs samples were processed in non-reducing conditions and denatured at 70°C for 10 min. For CD9^+^/CD63^+^/CD81^+^ EVs, CD81 and for Spike S1 was done in reducing conditions. Beads were removed from the denatured EV lysate using an Eppendorf magnet (Thermo Fisher) before SDS-PAGE. After transfer to nitrocellulose membrane (Amershan), blots were blocked in 5% low-fat milk and 0.1% Tween 20 O/N. After 3X washes in PBS- 0.1%-Tween 20, membranes were blotted with primary antibodies [anti-human CD81, Santa Cruz (sc-23962) 1/1000; anti-Spike RBS (Sino biological 40591-T62) 1/2000] for 1h followed by 3X washes in PBS-0.1% Tween. Blots were then incubated with a secondary anti-IgG mouse- IRD-800 1/15000 (LI-COR) or anti-IgG rabbit- IRD-680 for 1h followed by 3X washes in PBS-0.1% Tween. Fluorescence immunoblots were documented in an LI-COR ODYSSEY infrared system.

### Flow cytometry bead-based assay

SEC fractions from representative patients from G1, G2 and G3 were analyzed for the presence of CD9, CD63 and CD81 EVs markers by bead-based flow cytometry following a procedure previously described (Thery et al., 2006) and using the antibodies described in the Western blot section in addition to anti-human CD63, Immunostep (63PU-01MG) (dilutions: anti-CD9, anti-CD63 and anti-CD81 1/500).

### Protein extraction and digestion

#### CD9^+^ and mS1 captured EVs

Protein digestion was performed using the PreOmics kit (P.O.00001). Briefly, beads-EVs complexes were resuspended in 20 μl of PreOmics lysis buffer and incubated for 10 min at 1000 rpm and 70°C. Then, EVs lysates were loaded into the cartridges, and 20 μl of the PreOmics digestion solution was added to each sample. After incubating for 3h at 37°C, the enzyme reaction was stopped, several washes were performed, and the peptides were eluted with the elution buffer provided by the kit. Finally, peptides were completely dried in the speed-vac (45°C).

#### Human EVs isolated by SEC

SEC fractions were concentrated in a speedvac to reach a final volume of 250μl. The proteins were extracted by adding 750μl of 8M Urea/0.1M Tris-HCl pH8 with the help of a bioruptor (10 cycles of 30s ON/30s OFF). The process was done in ice in order to avoid the samples to heat. The samples were centrifuged at 12000 xg for 10 min at 4°C and the supernatants were recovered in new-clean tubes. The supernatants were precipitated with 250μl 100% trichloroacetic acid (TCA) (w:v) for 1h at 4°C. The samples were centrifuged at 12,000 xg for 10 min at 4°C and the resulting pellets were washed with 1ml of cold acetone at 4°C for 16h. The pellets were finally recovered by centrifugation at 12,000 xg for 10 min at 4°C and resuspended in 30μl of 6M Urea/0.1M Tris pH8 with the help of a bioruptor (10 cycles of 30s ON/30s OFF). The samples were then centrifuged at 20,000 xg for 10 min at 4°C to remove any insoluble material. Three micrograms of each sample were sequentially digested with LysC and trypsin. Prior to the digestion, the samples were reduced and alkylated with dithiothreitol (DTT) and Chloroacetamide (CAA) and diluted with 0.1M Tris-pH8 to reach a final concentration of 2mol/L of Urea. Lys-C was added at 1:25 (w/w; enzyme-to-proteins ratio) and the protein digestion was carried out at 30°C for 16h. The samples were diluted again with 0.1M Tris-HCl pH8 to reach a final concentration of 0.8mol/L of Urea. Trypsin was added at 1:25 (w/w; enzyme-to-protein ratio) and protein digestion was carried out at 30°C for 8h. Enzymatic reaction was stopped with 10% (v/v; final concentration). Samples were desalted using PolyLC C18 pipette tips according to the manufacturer instructions, dried in a speedvac and resuspended in 30ml of 0.1% (v/v) trifluoroacetic acid (TFA) before to be quantified in a nanodrop.

#### Hamster EVs isolated by SEC

Isolated EVs from hamster plasma were precipitated for 1h at -20°C using cold acetone. Samples were centrifuged at 13,000 xg for 15 minutes at 4°C. Supernatants were discarded, pellets air-dried and resuspended in 20 µl of PreOmics Lysis solution and processed as described above for CD9+ and mS1 EVs immunocaptured.

### Liquid chromatography mass spectrometry

#### CD9^+^ EVs (DIA)

The data was acquired with an Ultimate 3000 system (Thermo Fischer Scientific) coupled to an Orbitrap Exploris 480 mass spectrometer (Thermo Fischer Scientific) at the Hecklab (Utrecht University, Netherlands). Peptides were first trapped (Dr Maisch Reprosil C18, 3 µM, 2 cm × 100 µM) for 2 min at 300 nL/min in 9% buffer B (80% ACN, 0.1% FA) before separation on an analytical column (Agilent Poroshell, EC-C18, 2.7 µM, 50 cm x 75 µM). Peptide separation was performed at 300 nl/min for 95 min using a linear gradient of 13 to 44% B, followed by a 3 min steep increase to 99% B, a 5 min wash at 99% B and a 10 min re-equilibration step at 9% B. The mass spectrometer was operated in a data-independent mode (DIA). Peptides were ionized in a nESI source at 1.9 kV and focused at 40% amplitude of the RF lens. First, full scan MS1 spectra from 400 - 1000 m/z were acquired in the Orbitrap at a resolution of 60,000 with the AGC target set to 1×106 and for a maximum injection time of 100 ms. The MS1 scan was preceded by 30 sequential quadrupole isolation windows of 20 m/z that were fragmented by HCD, at 28% normalized collision energy, and MS2 scans from 145-1450 m/z were recorded in the Orbitrap at 30,000 resolution. For MS2 scans, the AGC target was set to 2 x 10^5^ under automated calculation of maximum injection time.

#### mS1 captured EVs (DIA)

Data was acquired using an LTQ-Orbitrap Fusion Lumos mass spectrometer (Thermo Fisher Scientific) coupled to an EASY-nLC 1200 (Thermo Fisher Scientific) at the Center for Genomic Research (Barcelona, Spain). Peptides were loaded directly onto the analytical column and were separated by reversed-phase chromatography using a 50-cm column with an inner diameter of 75 μm, packed with 2 μm C18 particles spectrometer (Thermo Scientific). Chromatographic gradients started at 95% buffer A and 5% buffer B with a flow rate of 300 nl/min for 5 minutes and gradually increased to 25% buffer B and 75% A in 105 min and then to 40% buffer B and 60% A in 15 min. After each analysis, the column was washed for 10 min with 10% buffer A and 90% buffer B. Buffer A: 0.1% formic acid in water. Buffer B: 0.1% formic acid in 80% acetonitrile. The mass spectrometer was operated in positive ionization mode with nanospray voltage set at 2.4 kV and source temperature at 305°C. The instrument was operated in data-independent acquisition mode. In each cycle of data-independent acquisition analysis, following each survey scan, 40 consecutive windows of 10 Da each were used to isolate and fragment all precursor ions from 500 to 900 m/z. A normalized stepped collision energy of 28% was used for higher-energy collisional dissociation (HCD) fragmentation. MS2 scan range was set from 350 to 1850 m/z with detection in the Orbitrap at a resolution of 30,000. Digested bovine serum albumin (New England Biolabs cat # P8108S) was analyzed between each sample to avoid sample carryover and to assure stability of the instrument and QCloud has been used to control instrument longitudinal performance during the project.

#### Human EVs isolated by SEC (DDA)

Data was acquired using a Advion TriVersa NanoMate (Advion) fitted on an Orbitrap Fusion Lumos™ Tribrid mass spectrometer (Thermo) at the Mass Spectrometry & Proteomics platform of the Institute for Research in Biomedicine (Barcelona, Spain). Peptides were diluted in 3%ACN, 1%FA. Samples were loaded to 300 μm × 5 mm C18 PepMap100, 5 samples were loaded to 300 μm × 5 mm C18 PepMap100, 5tein. 10% Thermo Scientific Dionex Ultimate 3000 chromatographic system (Thermo Scientific). Peptides were separated using a C18 analytical column (nanoEaseTM M/Z HSS C18 T3 (75 μm × 25 cm, 100Å, Waters) with a 150 min run, comprising three consecutive steps with linear gradients from 3% to 35% B in 120 min, from 35% to 50% B in 5 min, from 50% to 85% B in 2min, followed by isocratic elution at 85% B in 5 min and stabilization to initial conditions (A= 0.1% FA in water, B= 0.1% FA in CH3CN) at 250 nl/min flow rate. The mass spectrometer was operated in a data-dependent-acquisition (DDA) mode. Survey MS scans were acquired in the orbitrap with the resolution (defined at 200 m/z) set to 120,000. The lock mass was user-defined at 445.12 m/z in each Orbitrap scan. The top speed (most intense) ions per scan were fragmented by CID. The MSMS was detected in the Ion Trap (with Max Injection time of 35ms). The ion count target value was 400,000 for the survey scan and 10,000 (CID) for the MS/MS scan. Target ions already selected for MS/MS were dynamically excluded for 15s. Spray voltage in the NanoMate source was set to 1.70 kV. RF Lens were tuned to 30%. Minimal signal required to trigger MS to MS/MS switch was set to 5000 and activation Q was 0.250. The spectrometer was working in positive polarity mode and singly charge state precursors were rejected for fragmentation.

#### Hamster EVs isolated by SEC (DDA)

Samples from SARS-CoV-2 infected hamsters were processed as described above for human samples with slight modifications. Peptides were diluted in 3% ACN, 1% FA. Samples were loaded to a 300 μm × 5 mm Pep-Map C18 (Thermo Scientific) at a flow rate of 15 μl/min using a Thermo Scientific Dionex Ultimate 3000 chromatographic system (Thermo Scientific). Peptides were separated using a C18 analytical column (NanoEase MZ HSS T3 column, 75 μm × 250 mm, 1.8 μm, 100Å, Waters) with a 120 min run, comprising four consecutive steps, first 3 min of isocratic gradient at 3%B, from 3 to 35% B in 90 min, from 35 to 50% B in 5 min, from 50 to 85% B in 1 min, followed by isocratic elution at 85% B in 5 min and stabilization to initial conditions (A= 0.1% FA in water, B= 0.1% FA in CH3CN). The column outlet was directly connected to an Advion TriVersa NanoMate (Advion) fitted on an Orbitrap Fusion Lumos™ Tribrid (Thermo Scientific). The mass spectrometer was operated in a data-dependent acquisition (DDA) mode. Survey MS scans were acquired in the orbitrap with the resolution (defined at 200 m/z) set to 120,000. The lock mass was user-defined at 445.12 m/z in each Orbitrap scan. The top speed (most intense) ions per scan were fragmented in the CID and detected in the Ion Trap. Quadrupole isolation was employed to selectively isolate peptides of 350-1700 m/z. The predictive automatic gain control (pAGC) target was set to 4e5. The maximum injection time was set to 50ms for MS1 and 35ms for MS2. Included charged states were 2 to 7. Target ions already selected for MS/MS were dynamically excluded for 15 s. The mass tolerance of this dynamic exclusion was set to ±2.5 ppm from the calculated monoisotopic mass. Spray voltage in the NanoMate source was set to 1.7 kV. RF Lens were tuned to 30%. Minimal signal required to trigger MS to MS/MS switch was set to 5000 and activation Q was 0.250. The spectrometer was working in positive polarity mode and singly charge state precursors were rejected for fragmentation.

### Proteins identification and statistical analysis

#### CD9^+^ EVs (DIA)

Spectra were extracted from the DIA data using DIA-NN (version 1.7.15) and searched against the “Deep Learning” generated spectral library for the provided database (containing all reviewed human and SARS-CoV-2 protein sequences deposited in Uniprot and downloaded on June 2021). Trypsin was set as the digestion enzyme, for a minimum peptide length of 7 amino acids and a maximum peptide length of 30 amino acids and only one missed cleavage was tolerated. Cysteine carbamidomethylation was set as a fixed modification, and methionine oxidation was set as variable modification. N-terminal methionine excision was enabled. Precursor false discovery rate (FDR) was set to 1%, protein grouping was done by protein name and cross-run retention time dependent normalization was enabled. Label-free quantification was done, using unique peptides. The gene-centric report from DIA-NN was used for downstream analysis. Proteins identified in more than 85% of biological replicates of one condition but absent in the comparing condition, have been considered as absent/present. Human proteins have been filtered to remove keratins and proteins previously reported as contaminants of plasma-derived EVs proteins including immunoglobulins and complement proteins.

#### mS1 captured EVs (DIA)

Acquired spectra were analyzed using a library-free strategy with DIA-NN (Neural networks and interference correction enable deep proteome coverage in high throughput) (v1.7.15). The data were searched against a SARS-CoV-2 database and Swiss Prot Human database (April 2020). For peptide identification, trypsin was chosen as the enzyme and up to one missed cleavage was allowed. Oxidation of methionine was used as variable modifications whereas carbamidomethylation on cysteines was set as a fixed modification. False discovery rate (FDR) was set to a maximum of 1%. Precursor and fragment ion m/z mass range were adjusted to 400-1000 and 350-1850, respectively. Default settings were used for the other parameters. Proteins identified in more than 85% of biological replicates of one condition but absent in the comparing condition, have been considered as absence/presence. Human proteins have been filtered to remove keratins and proteins previously reported as contaminants of plasma derived EVs proteins including immunoglobulins and complement proteins.

#### Human EVs isolated by SEC

The data were searched against a database containing SP Human (January 2021) and SARS-CoV-2 (from NCBI), using the search algorithm Mascot v2.6 (http://www.matrixscience.com/). Peptides have been filtered based on FDR and only peptides with a minimum length of 7 amino acids and showing an FDR lower than 1% have been retained. Proteins identified in more than 66% of biological replicates in one condition but not in the compared condition, have been considered as absence/presence. Human proteins have been filtered to remove keratins and proteins previously reported as contaminants of plasma-derived EVs proteins including immunoglobulins and complement proteins.

#### Hamster EVs isolated by SEC

The data were searched against a database containing the reference proteome in Uniprot for Chinese hamster (UP000001075) and NCBI SARS-CoV-2, using the search algorithm Mascot v2.6 (http://www.matrixscience.com/). Peptides have been filtered based on FDR and only peptides with a minimum length of 7 amino acids and showing an FDR lower than 1% have been retained. Hamster proteins have been filtered to remove keratins and homologous proteins previously reported as contaminants of plasma derived EVs in human samples including immunoglobulins and complements.

Quantitative values of all the isolation methods have been normalized (global cross-run normalization) and Log_2_ transformed. For each of the group comparisons, a Fold Change, p-value and adjusted p-value (q value) have been calculated using the ‘limma’ package.

The mass spectrometry proteomics data have been deposited to the ProteomeXchange Consortium via the PRIDE partner repository (Perez-Riverol et al., 2019, Perez-Riverol et al., 2022) with the dataset identifier PXD041931”.

### Gene ontology analysis

Functional enrichment analysis of differentially expressed proteins in EVs isolated by DIC-CD9 immunocapture and mS1B were performed using DAVID Bioinformatics Resources 6.8 (Huang da et al., 2009).

## Results

### Study and experimental design

**Figure 1:**
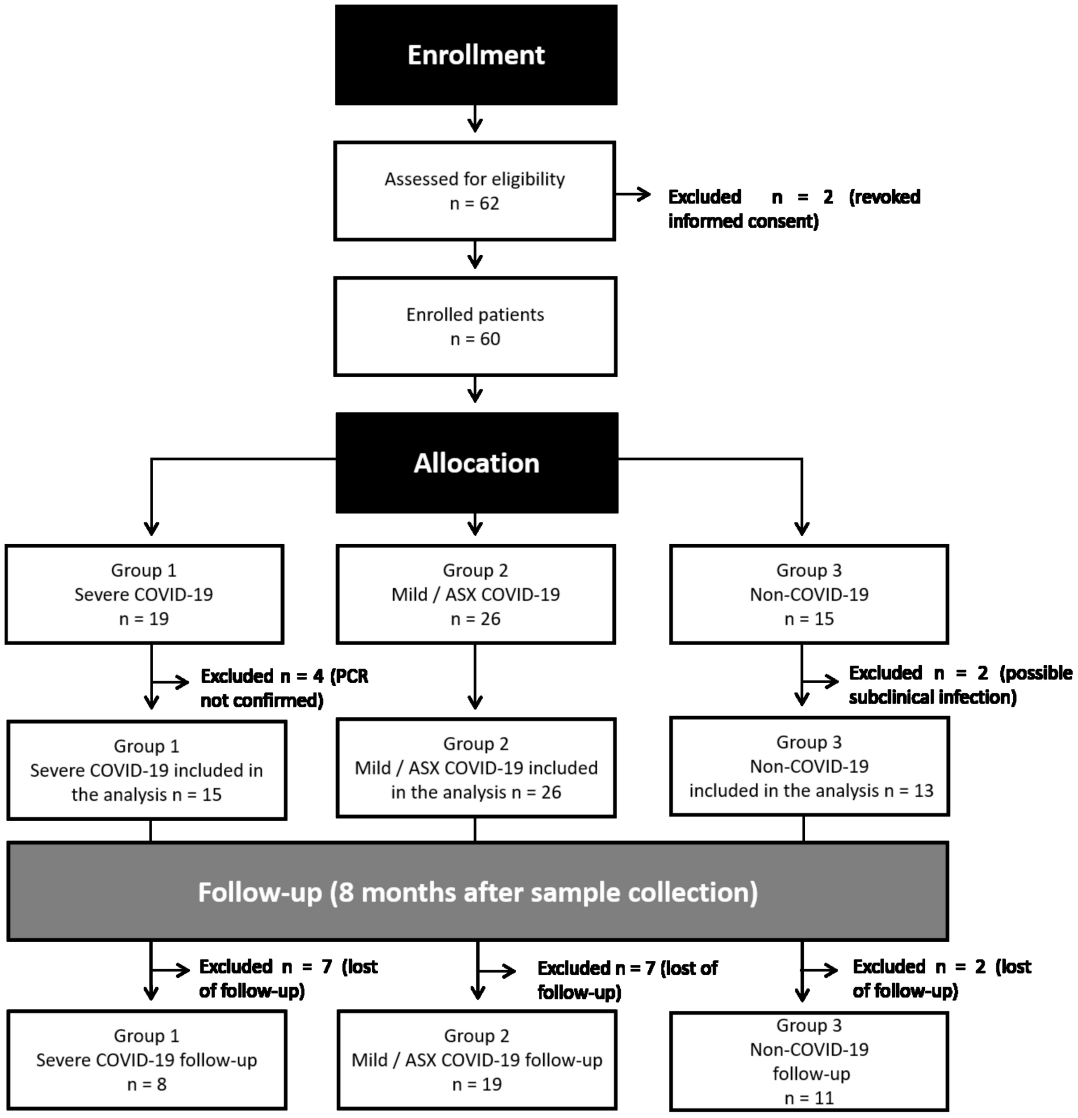
Flowchart of study design. After enrollment of 62 individuals in early 2020, two participants revoked informed consent and six did not meet the criteria of eligibility. Remaining samples were allocated to three different groups: Group 1 (G1, n=19), positive SARS-CoV-2 RT-PCR with severe clinical condition; Group 2 (G2, n=26), positive SARS-COV-2 RT-PCR with mild-symptoms related to SARS-CoV-2 and asymptomatic (ASX) individuals; Group 3 (G3, n=15) negative SARS-CoV-2 RT-PCR healthcare workers. An active follow-up visit was performed eight months after the initial recruitment for most of the individuals.

The study protocol was approved by the Ethics Committee of the Hospital Clinic of Barcelona (HCB/2020/0446). Following inclusion criteria and regarding the severity of the disease, patients were included in three groups: Group 1 (G1, n=19), positive SARS-CoV-2 RT-PCR with severe clinical condition independently of the organs involved. Group 2 (G2, n=26), positive SARS-CoV-2 RT-PCR with either asymptomatic or paucisymptomatic COVID-19, who did not require hospitalization nor specific treatment. Group 3 (G3, n=15) negative SARS-CoV-2 RT-PCR healthcare workers. Eight individuals were excluded from the study, two who revoked informed consent, and six that did not meet inclusion criteria. The study design and individual sample experiments are presented, respectively, in **Figure 1**, **Supplementary Figure 1** and **Supplementary Table 1**. Detailed clinical and demographic characteristics of the patients and uninfected volunteers included in the study can be found in the **Supplementary Table 2**.

### Clinical features of the patient’s cohort

The main clinical findings are summarized in **Table 1**. The median age of patients in G1 was older to that of patients in G2 and healthy volunteers in G3. No difference was observed in gender distribution between G1 and G2, whereas G3 was mostly composed of cisgender women. Of all clinical manifestations of COVID-19, symptoms such as respiratory insufficiency, expectoration, presenting a resting respiratory rate over 20 rpm, pulmonary infiltrates on chest X-ray, elevated transaminases and vomiting were exclusively reported in individuals belonging to G1. In contrast, several systemic symptoms were found in all groups of the cohort, but some had a significant difference between severe and mild/asymptomatic patients. Of interest, fever (p<0.005), coughing (p<0.005), dyspnoea (p<0.05) and coagulation or haemostasis disorders (p<0.05) were significantly more present in G1 than G2.

**Table 1:** Clinical data of participants included in this study.

	GROUP 1 SEVERE COVID-19 (N=15)	GROUP 2 MILD AND ASSX COVID-19 (N=26)	GROUP 3 NON COVID-19 (N=13)
**AGE (median, range)**	61.06 (24-94)	37.77 (23-73)	38 (24-65)
**SEX (total, %)** Male Female	9 (60) 6 (40)	9 (35) 17 (65)	1 (7.7) 12 (9.3)
**SYMPTOMATOLOGY (TOTAL, %)** Fever Headache Asthenia Anorexia Myalgia Coughing Expectoration Chest Pain Dyspnoea Rhinorrhoea Odynophagia Anosmia Ageusia / Dysgeusia Nausea Vomiting Diarrhoea Skin disorders Elevated transaminases Coagulation / Haemostasis disorders Respiratory insufficiency Resting respiratory rate >20rpm Pulmonary infiltrates on chest X-Ray Fever maintained in the last 72h Suggestive COVID-19 Rx or radiological progression	11 (7.3) 3 (20) 7 (46.67) 3 (20) 4 (26.67) 10 (66.67) 2 (13.3) 0 9 (60) 1 (6.67) 2 (13.33) 0 4 (26.67) 3 (20) 2 (13.33) 3 (20) 0 2 (13.33) 3 (20) 10 (66.67) 6 (40) 14 (93.33) 7 (46.67) 11 (73.33)	5 (19.23) 9 (34.6) 9 (34.6) 4 (15.38) 3 (11.53) 5 (19.23) 0 2 (7.6) 6 (23) 5 (19.23) 2 (7.6) 2 (7.6) 5 (19.23) 1 (3.8) 0 1 (3.8) 1 (3.8) 0 0 0 0 0 1 (3.8) 1 (3.8)	0 3 (23.1) 2 (15.38) 0 1 (7.6) 0 0 2 (15.38) 3 (23.1) 2 (15.38) 0 1 (7.6) 0 0 0 0 0 1 (7.6) 0 0 0 0 0
**COMORBIDITIES (TOTAL, %)** HTN DM Severe kidney disease: transplant Severe kidney disease: haemodialysis Neoplasia COPD Cardiovascular disease Chronic liver disease Immunosuppressants Others	8 (53.33) 2 (13.33) 0 1 (6.67) 1 (6.67) 2 (13.33) 4 (26.67) 0 1 (6.67) 9 (60)	3 (11.53) 3 (11.53) 0 0 1 (3.8) 0 1 (3.8) 0 0 11 (42.3)	3 (23.1) 0 0 0 0 1 (7.6) 0 0 0 2 (15.38)
**MORTALITY (TOTAL, %)**	1 (6.7)	0	0
**FOLLOW-UP SAMPLES (TOTAL, %)**	8 (53.33)	19 (73)	11 (84.6)
**VACCINATION AT FOLLOW-UP (TOTAL, %)**	0	7 (26.9)	7 (53.8)

Regarding comorbidities, the most common chronic diseases in our cohort were hypertension (HTN) (25.9%), diabetes mellitus (DM) and cardiovascular disease (both 9.2%). Of note, we found that G1 presented a statistically significant difference of HTN comorbidity when compared to G2 (p<0.05). We also observed an association between severity of COVID-19 pathology and the number of comorbidities identified between groups. Unfortunately, one of the 54 patients included in the study died. This patient, enrolled in G1, was an elderly patient with concomitant comorbidities such as HIV infection, severe nephropathy, cardiovascular disease and HTN.

### Clinical laboratory data

Clinical laboratory data collected from all patients enrolled in the study showed that C-Reactive protein (CRP) levels increased with disease severity, together with D-dimer, Ferritin, lactate dehydrogenase (LDH), Gamma-glutamyl transferase (GGT), alkaline phosphatase, procalcitonin, ferritin, monocytes and hypochromic RBCs. Several variables decreased with progression of disease severity: cholesterol, total protein, albumin, calcium and basophils frequency (**Supplementary Figure 2**, **Supplementary Table 3**). Despite all prognostic severity values found in the clinical laboratory data, we did not find any significant marker of infection as G2 and G3 had no significant difference in any of these values. Yet, significant differences were observed in between-group comparisons of clinical parameters (**Supplementary Figure 2**).

### Humoral immune response

**Figure 2:**
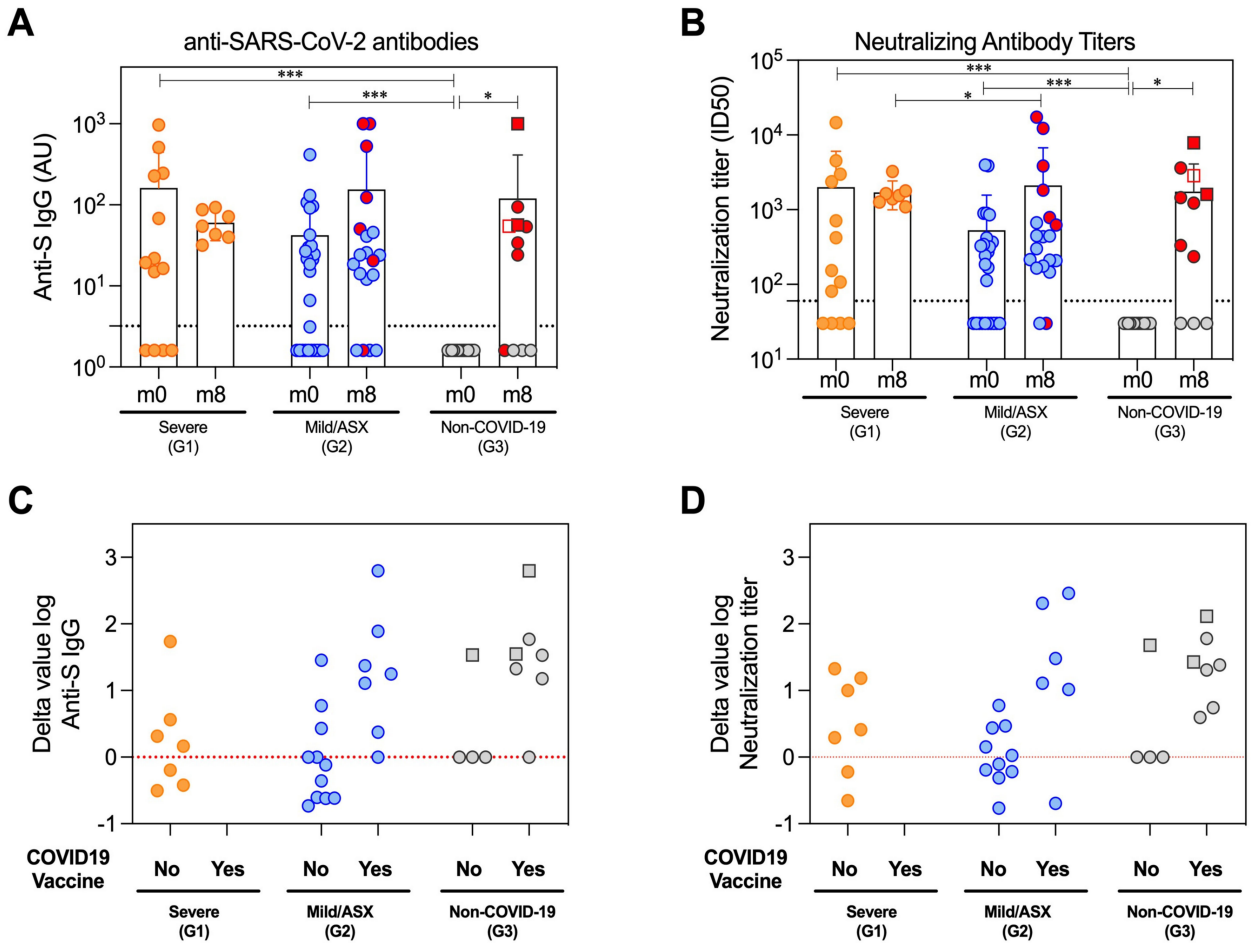
Humoral immune response of the patient’s cohort. Antibodies profiles at beginning of the study (m0) and eight months later (m8) in the study groups severe (G1), mild and asymptomatic (G2) and non-COVID-19 hospital care workers (G3). **(A)** Total reactive IgG antibodies against the S protein of the SARS-CoV-2 measured by an in-house ELISA. The lower limit of detection (dashed line) of the assay was 3.2. All Ig levels (UA) without value but measured by ELISA were plotted as 1.6 (average of 0 and 3.2 AU/ml that is lower limit of detection). **(B)** Neutralization assay antibody titers against the wild-type SARS-CoV-2 pseudoviruses in serum samples. The lower limit of detection (dashed line) of the assay was 60. All nAbs levels with ID50<60 were plotted as 30 (average of 0 and 60 that is lower limit of detection). **(C, D)** Difference in the log value of anti-S IgG and neutralizing antibodies between the last sampling time (m8) and peak of the infection (m0) depending on the study group (G1/severe, G2/Mild/ASX and G3/Non-COVID-19) and COVID-19 vaccination. Red line represents stable antibody titers throughout the study. The circles represent individual participants, and the bars mean with SD. Individuals from G3 infected between m0 and m8 are denoted with squared symbols. Vaccinated individuals are highlighted with red-filled symbols in **(A, B)** Longitudinal comparisons for each group were tested using Wilcoxon signed rank test. Comparisons between groups in each time point were assessed by Kruskal-Wallis and Conover’s post-hoc tests. p-values: *p<0.05, ***p<0.001 in **(A, B)**.

The generation of specific antibodies against SARS-CoV-2 confirms the viral infection and indicates the development of the humoral adaptive immune response elicited to try to counteract the COVID-19. Therefore, we measured antigen-specific antibody levels. Subclassing immunoglobulins (IgG, IgA and IgM) were determined against the viral Spike (S) and nucleocapsid (N) proteins at the initial time of infection (m0) and 8 months after (m8) in all study groups. Likewise, we measured the neutralizing capacity of antibodies at both time points. The overall percentage of individuals with detectable antibody levels against the Spike protein during acute infection (m0) was 69% IgG, 69% IgA and 69% IgM in G1; 62% IgG, 69% IgA and 54% IgM in G2; and no detectable levels of any immunoglobulin class in G3 (**Figure 2A**, **Supplementary Table 4**). Similar levels of neutralization antibody titers suggested the functionality of the detectable anti-Spike IgG (**Figure 2B**). Detection of Ig subclass levels against N protein at the same time point was 62% IgG, 54% IgA and 38% IgM in G1; 58% IgG, 15% IgA and 23% IgM in G2; and no detectable levels in G3, except for one healthy volunteer who surprisingly showed IgM antibodies (**Supplementary Figure 3**). Eight months later (m8), all cases that remained in G1 had detectable S-specific IgG and neutralizing responses, a greater number in G2 partially due to vaccination, and 53,8% of G3 subjects explained by either vaccination (n=4) or new infection (n=3) (**Figures 2A-D**). Interestingly, IgG levels and neutralization titers against S during acute infection (m0) were comparable between G1 and G2, remaining most of them similarly detectable 8 months later. These antibody levels were additional evidence of SARS-CoV-2 infection in these individuals and proof of immune response against the virus or the subsequent vaccination (**Figure 2**, **Supplementary Figure 3**, **Supplementary Table 4**).

### Isolation, characterization and proteomic analysis of circulating EVs during active COVID-19 infection

We developed an exhaustive pipeline to detect viral peptides associated with EVs in the plasma of COVID-19 patients using MS (**Supplementary Figure 1**). Moreover, in order to analyze the presence of EV-markers, we customized a database that compiled 85 EV proteins identified by proteomics in human plasma samples isolated by size-exclusion chromatography (SEC) (de Menezes-Neto et al., 2015), density gradient centrifugation (Kugeratski et al., 2021) and in different EVs subpopulation obtained by CD9, CD81 and CD63 immunocapture (Kowal et al., 2016; Jeppesen et al., 2019) (**Supplementary Table 5**). Our first approach relied on the knowledge that CD9 is a tetraspanin considered a canonical marker present in EVs from all types of biofluids, including plasma (Mizenko et al., 2021), and recently identified in EVs from patients using imaging flow cytometry (Tertel et al., 2022) or immuno-TEM (Kawasaki et al., 2022). Circulating CD9^+^-EVs from COVID-19 patients were initially isolated by a two-step purification procedure involving 120,000 *xg* ultracentrifugation of plasma samples that generate p120 fractions followed by CD9^+^-EVs magnetic immunocapture. Western-blot analysis of immunocaptured fractions showed a substantial increase in CD9 and CD81 signal in all samples when compared to equivalent protein amounts of p120 fractions used as input, indicating successful CD9^+^-EVs enrichment (**Supplementary Figure 4**). To identify SARS-CoV-2 peptides, we performed label-free liquid chromatography with tandem mass spectrometry (LC-MS/MS) analysis of EVs isolated by CD9 direct immunocapture at the peak of infection. Our analysis did not detect any viral peptide associated with circulating CD9^+^-EVs in any of the groups (**Supplementary Table 6**).

It has been shown that EVs contain gangliosides in their membranes (de Gassart et al., 2003). Thus, we developed a new EVs isolation methodology based on the ganglioside’s binding capacities of Siglec-1/CD169 receptor, a member of the immunoglobulin superfamily that binds to glycoconjugate ligands on cell surfaces in a sialic acid-dependent manner (Perez-Zsolt et al., 2021). Our new EV isolation platform is based on the specific interaction between CD169 protein and gangliosides dragged in the membrane of EVs (Perez-Zsolt et al., 2019). For this analysis, we employed latex beads coated with a recombinant protein named mS1 that includes a truncated form of Siglec-1 containing the sialylated ligand-binding domain. We isolated EVs from total plasma from seven severe COVID-19 patients (G1) and five non-COVID-19 individuals (G3). Proteomic analysis of the mS1-bound EVs indicate the presence of 69 EV markers, confirming the effective capture of plasma-derived EVs using this approach. However, inspection of proteomic data for presence of SARS-CoV2 derived peptides in total EVs trapped with mS1-beads, did not detect viral peptides in plasma from COVID-19 patients (**Supplementary Table 7**).

In a third attempt to identify viral peptides in circulating EVs, we performed SEC, a classical technique that has previously identified viral peptides in circulating EVs from a single-stranded RNA virus causing porcine respiratory distress (Montaner-Tarbes et al., 2016, 2018). For that, we employed a commercial system consisting of qEV 10 ml sepharose 2B columns and an automated fraction collector (iZon Sciences) for the processing of plasma from three individuals of each group. Molecular characterization of SEC fractions by bead-based flow cytometry analysis showed the enrichment of EVs containing CD9 tetraspanin in F3-F4 according to EVs profiles reported by the manufacturers, further validated by protein concentration quantification that showed separation of EVs from soluble proteins present in distal SEC fractions (F7-F10). NTA analysis of pooled F3-F5 fractions from representative patients of each group showed sizes and distribution compatible with EVs (**Supplementary Figure 5**). Mass spectrometry analysis of EVs isolated by SEC, one more time, did not detect SARS-CoV-2 peptides in total circulating EVs from COVID-19 patients (**Supplementary Table 8**).

Of interest, we were able to compare the results of MS obtained from the three different methodologies in one patient (ECO12). As shown in **Supplementary Figure 6**, the main findings observed in the MS analysis from all patients and healthy controls are also observed in this patient: (i) the number of proteins identified is highest in DIC CD9+-EVs, followed by ms-1 captured EVs and SEC; (ii) the Venn diagram confirms that they are complementary isolation techniques with a core of common proteins and unique proteins pertaining to each isolation method; (iii) GO analysis of the core proteins confirmed that they correspond to extracellular exosome and blood microparticles.

Finally, we employed an *in vivo* model using SARS-CoV-2 infected hamsters as a system to extend our search for viral proteins or peptides in circulating EVs (Brustolin et al., 2021). For this purpose, Syrian hamsters were infected with SARS-CoV-2 intranasally and RT-PCR analysis of upper and lower respiratory tracts (nasal turbinates and lungs) at 2, 4 and 7 dpi, confirmed active SARS-CoV-2 replication. MS analysis of hamsters’ plasma-derived EVs isolated by SEC detected an overall of 140 proteins including homologous to human EVs markers (ACTB, EZR, PYK, ALD, GAPDH, HLA1, RAP1B) (**Supplementary Figure 7**), indicating the presence of EVs in SEC fractions. Noticeably, this analysis did not detect SARS-CoV-2 peptides in this model.

**Figure 3:**
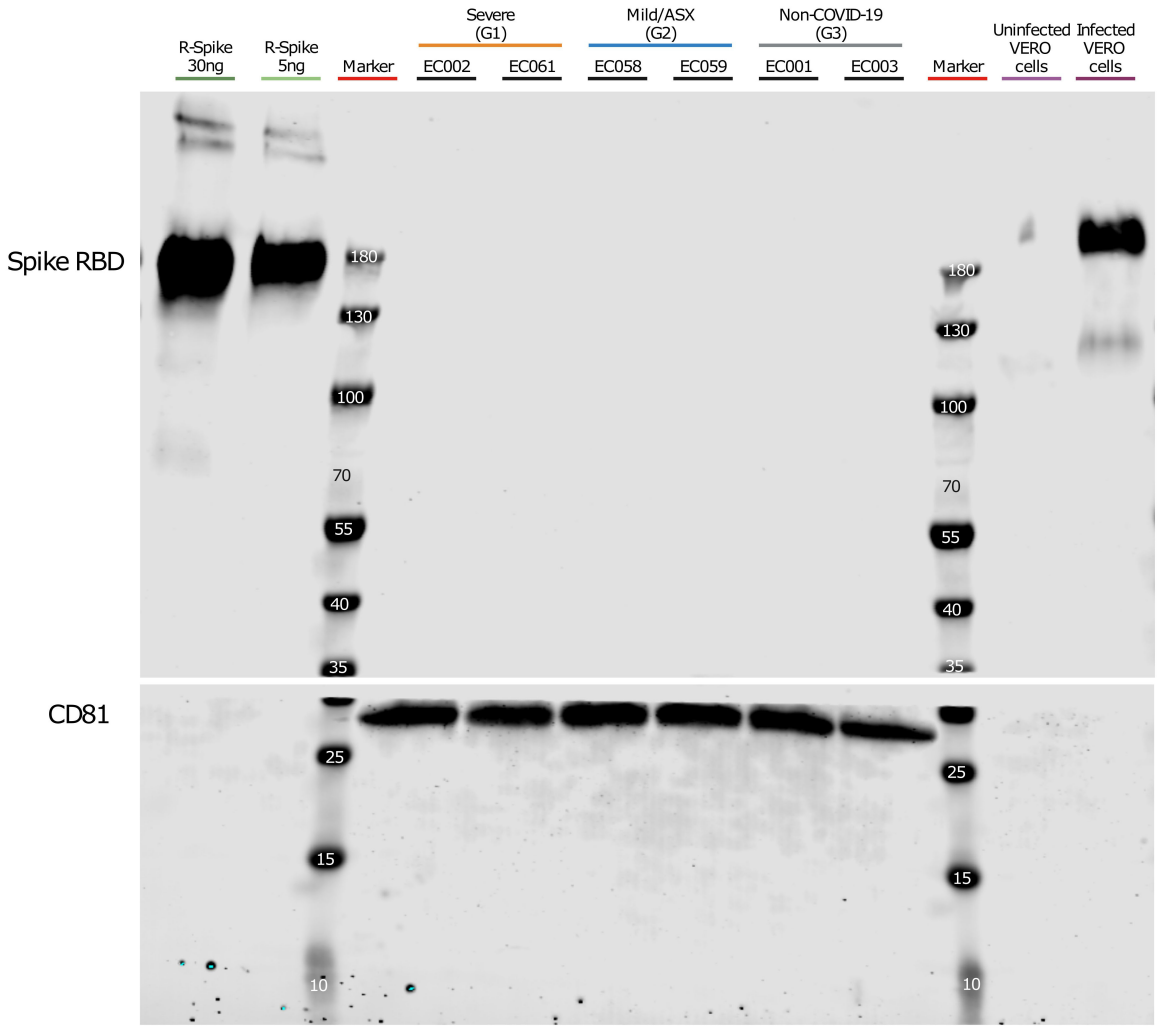
Western blot analysis of CD9^+^/CD63^+^/CD81^+^ EVs. Plasma from severe, Mild/ASX COVID-19 patients and non-COVID-19 individuals was used to immunocapture CD9^+^/CD63^+^/CD81^+^ EVs. Immunocaptured EVs were analyzed for the presence of SARS-CoV2 Spike Receptor binding domain (RBD). The membrane was cut at 35kDa and an anti-CD81 antibody was used as a control of quality and quantity of EVs. Recombinant Spike protein (30ng and 5ng) and a cell lysate of Vero cells uninfected and infected with SARS-CoV-2 were used as controls for S-RBD detection.

Our findings were remarkable, especially considering a report that detected the Spike protein using different technologies including Western-blot analysis after enriching CD9/CD81/CD63 EV subpopulations in COVID-19 patients (Pesce et al., 2021). Therefore, we performed a similar EV isolation based in CD9-CD63-CD81 immunocapture in samples from a subset of patients from each group (see materials and methods) followed by Western-blot analysis using anti-Spike RBD antibodies. As positive controls, we used Vero cells infected with SARS-CoV-2 and a recombinant protein containing SARS-CoV-2 Spike-RBD domain. Prior to the Western blot analysis, we performed Western-blot of infected and uninfected Vero cells, as well as recombinant R-Spike protein to ensure correct antibody detection (data not shown). As shown in **Figure 3**, we could not detect the S protein in the CD9/CD81/CD63 EV-enriched samples whereas the recombinant S-RBD of SARS-CoV-2 showed a positive signal as well as the control cell lysate of infected Vero cells with SARS-CoV-2 virus. Canonical EVs marker CD81 was detected in the same membrane in the EasySep EVs preparations. Last, we generated MS data of 300 ng and 30 ng of the S-protein (Sino Biological) mixed with 300 μl of the EV-enriched fraction from patient EC003 (proteomics analysis from these same fractions of this patient did not detect S-protein (**Supplementary Table 9**). As shown in **Supplementary Table 9**, S-protein peptides were readily and robustly detected. Moreover, we also demonstrated that 3 ng of S-proteins are also readily detectable by MS (**Supplementary Table 9**).

Collectively, mass spectrometry-based proteomics was performed on circulating EVs from COVID-19 patients with varying clinical outcomes, isolated through three different methods. Additionally, proteomic analysis was conducted on an *in vivo* model using SARS-CoV-2-infected hamsters, and Western blot analysis targeted the SARS-CoV-2 S-RBD domain. These combined approaches strongly suggest that SARS-CoV-2 viral peptides are not found in association with circulating EVs in COVID-19 patients.

### Molecular EV-signatures in COVID-19 patients with different clinical manifestations

**Figure 4:**
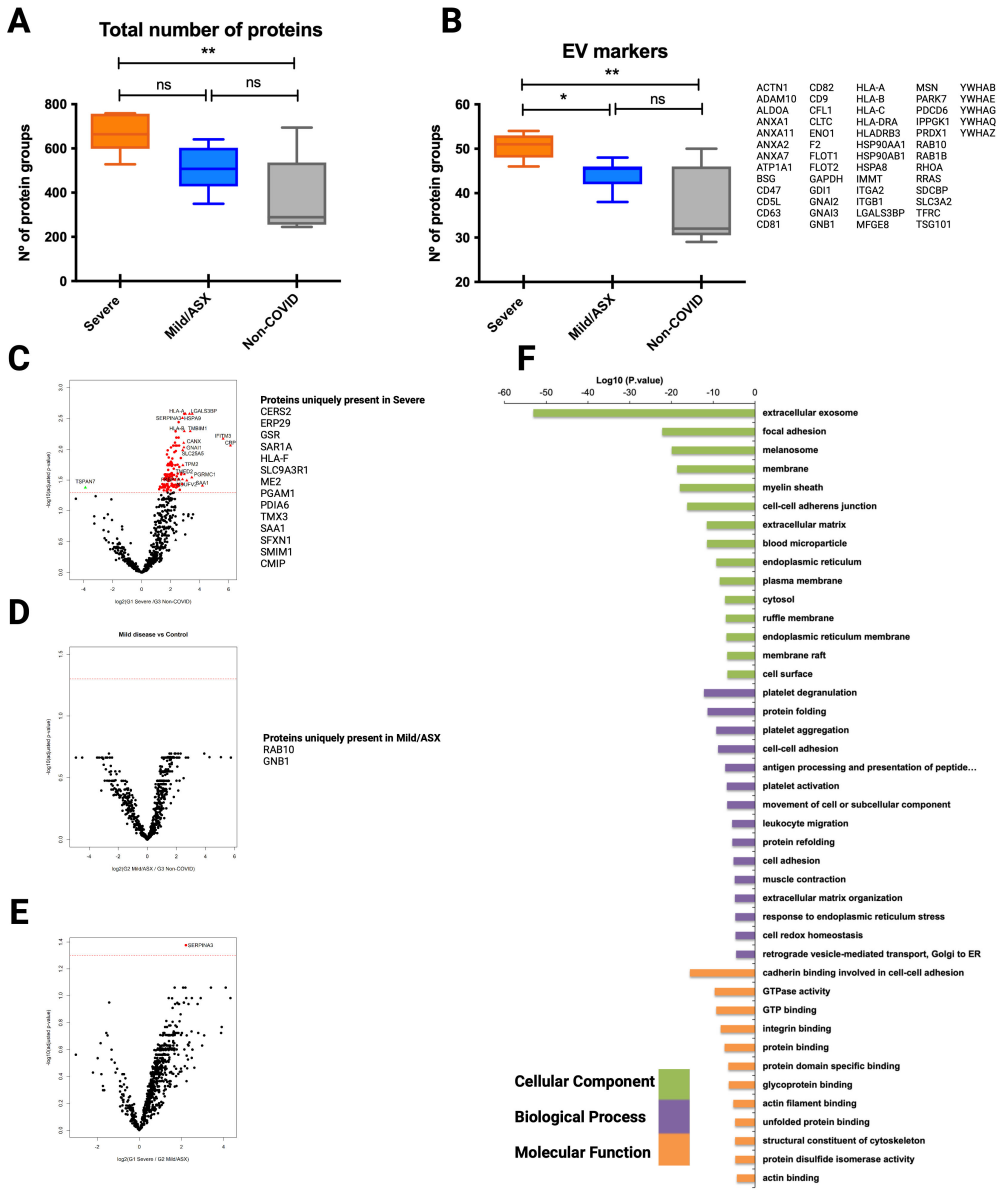
Proteomic analysis of CD9^+^ EVs from COVID-19 patients isolated by immunocapture at the infection peak. Seven individuals from G1, ten from G2 and 5 from G3 were used for CD9+ plasma-derived EVs isolation by magnetic immunocapture. Immunocaptured EVs were digested and analyzed by LC-MS/MS mass spectrometry. **(A)** Distribution of total number of proteins identified in each group at the infection peak. **(B)** Distribution and identity of the EV markers identified in the proteome from each cohort group. Statistical differences were tested using a One-way ANOVA (Kruskal Wallis post-test) with Dunn’s multiple comparisons. **(C-E)** CD9+ EVs protein abundance from each infected group was compared to abundance in the non-COVID-19 group. Volcano plot representation highlighting proteins up and down-regulated with statistical significance (q-val <0.05) (dotted red line). Proteins listed aside each plot correspond to proteins uniquely identified in each group. **(F)** Functional enrichment analysis by Gene Ontology (GO) of protein differentially present in CD9+ EVs from Severe COVID-19 patients when compared to non-COVID-19 individuals. Plots show the top enriched terms for Biological processes, Cellular Components and Molecular Functions with an FDR <0.05. *p value <0.05, **p value <0.005, ns, no-significant

Beyond the original hypothesis, the set of EV-associated human proteins identified during different stages of the infection should provide additional, valuable descriptive proteomic information. Thus, we explored the human proteome of EVs isolated by the three different methodologies. In the mass spectrometry of CD9^+^-EVs we identified an overall of 897 human protein groups from all samples (**Supplementary Table 6**). Interestingly, the total number of proteins identified from G1 severe patients was significantly higher than the total number of G3 non-COVID-19 individuals used as controls, while no differences were observed when compared to G2 mild/asymptomatic patients (**Figure 4A**). We identified 72 EV markers (**Figure 4B**) including the tetraspanins CD9, CD63, CD81, several members of the annexin family, isoforms of major histocompatibility class I molecules, TSG101, Flotillin 1 and 2, some RAB proteins and the recently described universal exosomal marker syntenin-1 (Kugeratski et al., 2021), among others. These results indicate a high enrichment of EVs using this methodology. The total number of EVs markers was also found significantly increased in severely affected patients when compared to both mild/asymptomatic patients and non-infected subjects, suggesting an increased amount of circulating CD9^+^-EVs in severe COVID-19 individuals.

Volcano plots illustrating statistical comparison of the abundance of human proteins detected in CD9^+^-EVs from severe COVID-19 patients and non-COVID-19 individuals showed 140 proteins differentially upregulated in infected patients with severe symptoms (q val= 0.05) (**Figure 4C**). Precisely, 14 of these were uniquely present in severe SARS-CoV-2 infection. Interestingly, when abundance of CD9^+^-EVs from mild/asymptomatic patients was compared to control subjects, no significant differences were observed (**Figure 4D**), except for Rab-13 and GNB1 two previously identified EVs GTPases involved in regulation of membrane trafficking of endosomal compartments and in signal transduction which were upregulated in mild/asymptomatic patients. These results indicate that a mild/asymptomatic SARS-CoV-2 infection does not induce major pathological alterations that translate to changes in the cargo and amount of circulating CD9^+^-EVs. Although the abundance of many proteins was greater in the most severely affected patients when compared to mild/asymptomatic, these differences were not statistically significant (**Figure 4E**). Gene ontology functional enrichment analysis of upregulated proteins showed that the most significantly represented cell compartment GO term corresponds to extracellular exosomes indicating the vesicular origin of these protein subset (**Figure 4F**). Biological function enriched GO terms are related to platelets degranulation, aggregation and activation, protein folding, cell-cell adhesion, antigen processing and presentation, leukocyte migration, response to endoplasmic reticulum and stress, among others, indicating signatures of active inflammatory immune response and cellular stress. Together, these results suggest that SARS-CoV-2 induces pathological changes in severely affected individuals that are responsible for profound remodeling in the protein cargo of circulating CD9^+^-EVs reflecting ongoing inflammation, active immune response and cellular stress.

**Figure 5:**
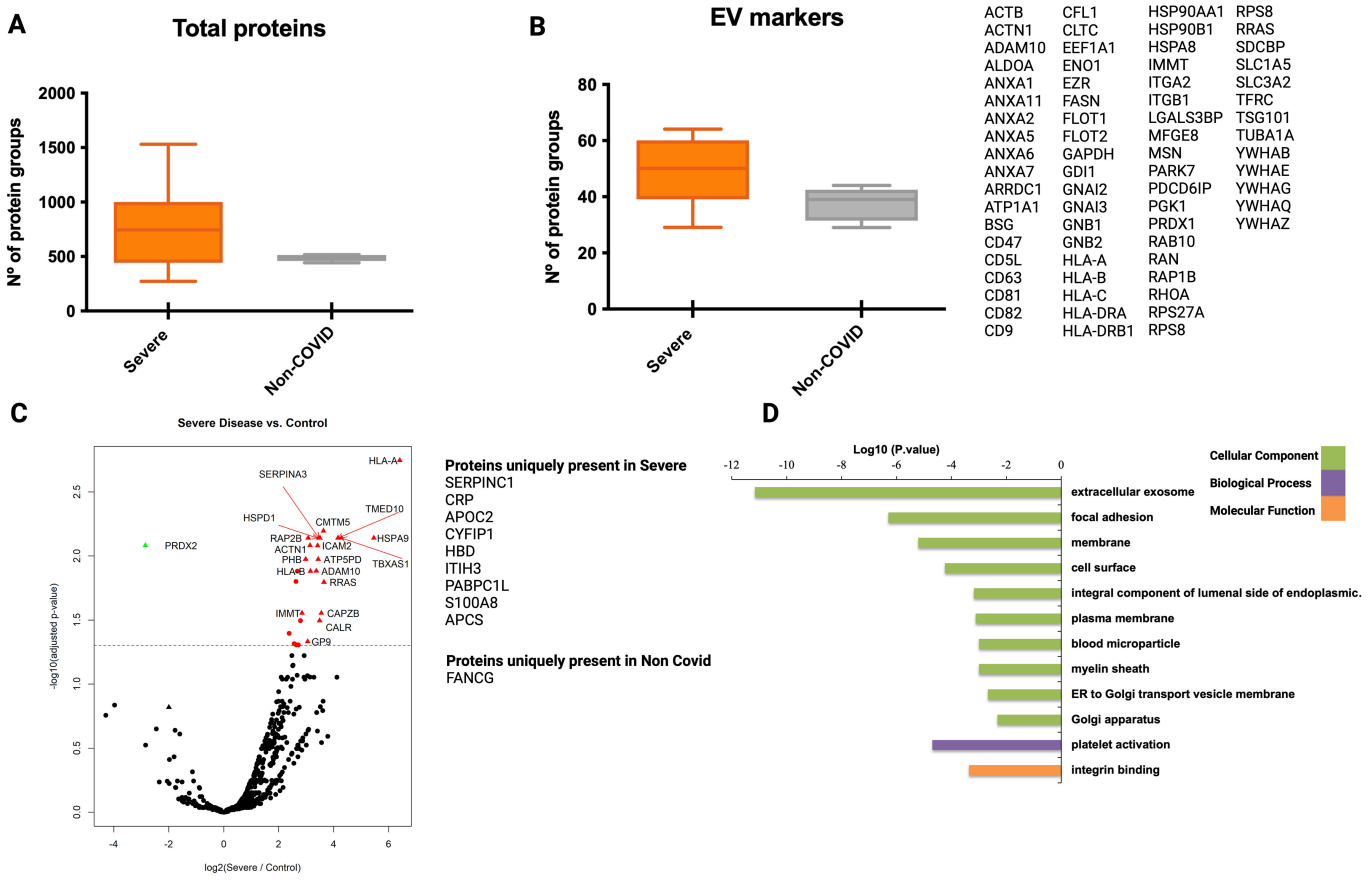
Proteomic analysis of plasma-derived EVs from COVID-19 patients isolated by mS1 mediated capture at the infection peak. Plasma from seven individuals from G1 and five from G3 at the peak of infection were used for isolation of EVs using mS1 mediated capture followed by LC-MS/MS. **(A)** Distribution of total number of proteins identified in isolated EVs. **(B)** Distribution and identity of the EV markers in the proteome of the analyzed groups. Statistical significance was evaluated by a t-test (Mann Whitney). **(C)** EVs protein abundance from severe COVID-19 and non-COVID-19 individuals were compared. Volcano plot highlighting proteins found up and down-regulated in severe COVID-19 patients with statistical significance (q-val <0.05). Proteins listed aside correspond to proteins uniquely identified in each group. **(D)** Functional enrichment analysis by GO of protein differentially present in mS1-captured EVs from severe COVID-19 patients when compared to non-COVID-19 individuals. Plots show the top enriched terms for Biological processes, Cellular Components and Molecular Functions with an FDR <0.05.

When analyzing the human proteome of EVs isolated by mS1-capture, we did not observe statistically significant differences in the number of total proteins and EV markers identified in severe COVID-19 patients versus non-COVID-19 individuals (**Figures 5A, B**). However, when we compared the abundance of the proteins detected in both groups, we found 46 differentially expressed and 13 unique proteins present in severe patients (**Figure 5C**). Similar to what we found in the CD9^+^-EVs, gene ontology functional analysis of the differentially expressed protein in mS1-captured EVs show the enrichment of extracellular exosome terms in the category of cellular components (**Figure 5D**). Importantly, 13 out of the 46 differentially enriched proteins in mS1-captured EVs were also detected in CD9^+^-EVs including SERPINA3, IFITM3, HLA-A and HLA-B. Of note, we observed that out of the 1286 total proteins identified in mS1-captures EVs, a subset of 171 proteins including 26 classical EV markers were detected in negative controls where mS1-latex beads were incubated with PBS indicating a background saturation of mS1 recombinant protein with HEK293T cells secreted EVs in the mS1 production step.

Our analysis of EVs isolated from plasma of 12 COVID-19 patients by SEC at the peak of infection identified 451 protein groups in all the patients analyzed (**Supplementary Table 8**). Comparison of the total quantified proteins among the groups showed no significant differences, although patients with severe pulmonary disease in G1 presented a tendency to higher number of total proteins when compared to other groups (**Supplementary Figure 8A**). From this list, 46 EVs markers were detected in patients from all groups with a homogenous distribution among them (**Supplementary Figure 8B**, **Supplementary Table 8**). Except for PIGR receptor [two-fold increased association with EVs from G2 (q-value <0.1)], the abundance of other human proteins identified in plasma-derived EVs from G1 and G2 infected groups when compared to those present in EVs in G3 did not show statistically significant differences (**Supplementary Figure 8C**). Yet, we identified a set of proteins that were exclusively associated with SARS-CoV-2 infection. These included CRP, ENO3, FAH and FLC, proteins involved in inflammation, carbohydrate and amino acids metabolism and iron homeostasis, respectively.

### Molecular signatures of COVID-19 progression

**Figure 6:**
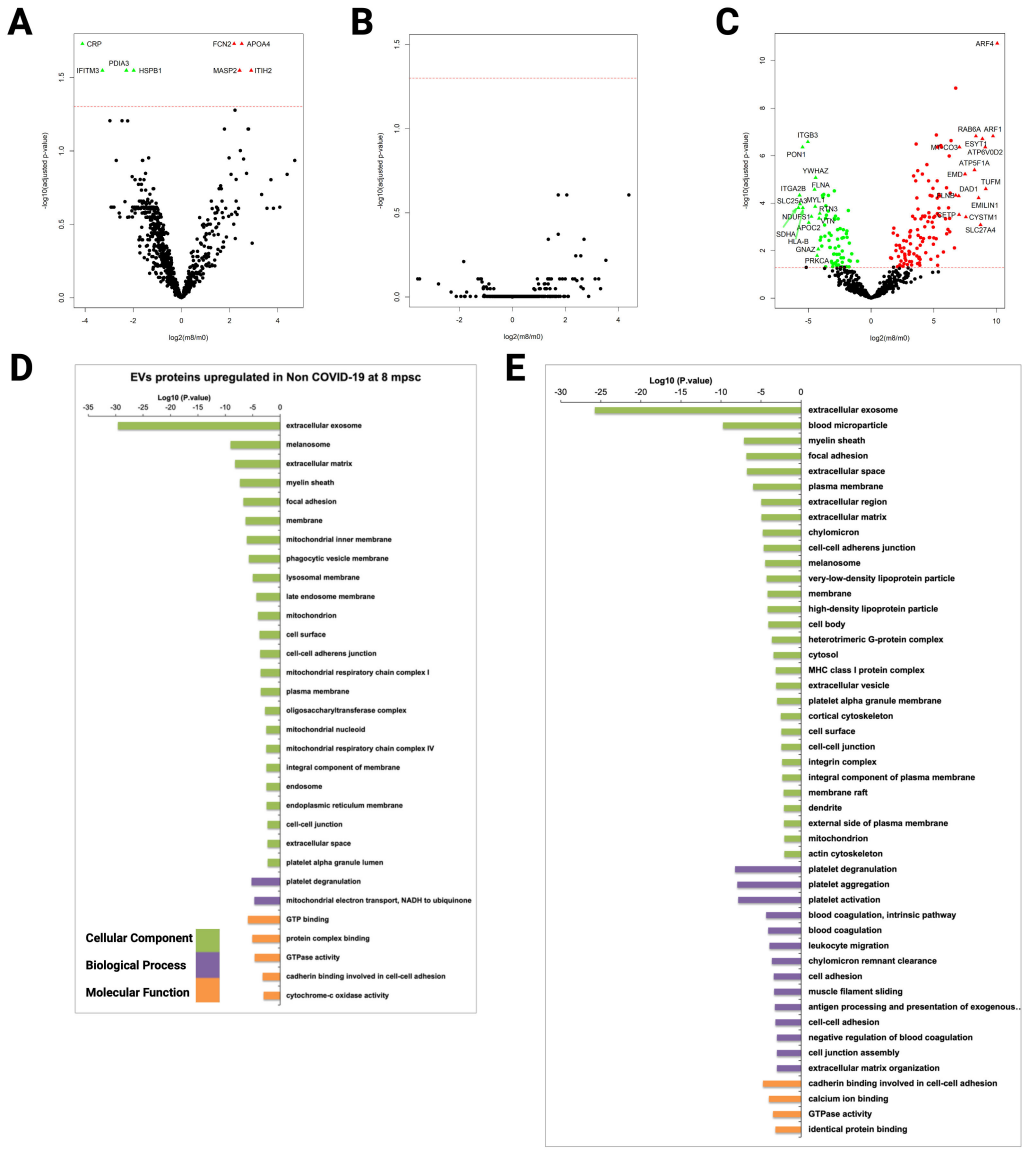
Proteomic analysis of CD9+ plasma-derived EVs from COVID-19 patients isolated by immunocapture at early infection and 8 months thereafter. Samples from 7 individuals from G1, 10 from G2, and 5 from G3 at early infection (m0) and 8 months thereafter (m8) were used for CD9^+^ plasma- derived EVs isolation via magnetic immunocapture. Immunocaptured EVs were digested and analyzed by LC-MS/MS. **(A–C)** CD9^+^-EVs protein abundance from each infected group at early infection was compared to the abundance 8 months later. Volcano plots show proteins significantly upregulated or downregulated (q-val < 0.05): **(A)** Severe, **(B)** Mild/ASX, and **(C)** Non-COVID. **(D, E)** Functional enrichment analysis by GO of proteins upregulated **(D)** and downregulated **(E)** in CD9^+^ EVs from non-COVID-19 individuals (G3) at the first time point compared to 8 months thereafter. Plots show the top enriched terms for Biological processes, Cellular Components and Molecular Functions with an FDR <0.05.

To identify protein signatures on EVs that reflect active COVID-19 infection and to distinguish them from convalescent state, we compared the proteome of patients CD9^+^-EVs from all groups eight months after the peak of the infection (m8) (**Supplementary Table 6**). The total number of proteins identified in CD9^+^-EVs showed a slight decrease in severe COVID-19 patients at the convalescent stage (**Supplementary Figure 9A**) and remained unchanged in mild/asymptomatic COVID-19 and control group (**Supplementary Figures 9B, C**). Interestingly, differential expression analysis showed that although a great number of proteins showed upregulation in CD9^+^-EVs of severely affected individuals at the infection peak (m0), only eight proteins were significantly different (four upregulated and four downregulated) in the convalescent state, suggesting that after symptoms remission, CD9^+^-EVs cargo in severe COVID-19 remain similar to that of the acute infection peak (**Figure 6A**). This observation would suggest that SARS-CoV-2 may lead to long-term pathological changes in patients with severe disease which are reflected in the circulating CD9^+^-EVs cargo. On the other hand, convalescent mild/asymptomatic subjects did not show significant changes in CD9^+^-EVs protein abundance when comparing m0 versus m8 (**Figure 6B**). This agrees with the absence of changes also observed in CD9^+^-EVs content of this group at m0 when compared to non-COVID-19 individuals, confirming that circulating CD9^+^-EVs are not altered by mild/asymptomatic SARS-CoV-2 infection.

Strikingly, in our analysis of non-COVID-19 health care workers (G3) we found that after 8 months post-sample collection there were statistically significant changes in the abundance of 194 proteins associated to CD9^+^-EVs when compared to the primary time point (124 up and 70 downregulated, **Figure 6C**). This result could be attributed to the fact that 80% of the subjects of this group had already received one dose of SARS-CoV-2 vaccine before m8 sample collection; therefore, CD9^+^-EVs cargo could be altered due to the immune response triggered by the vaccine. Gene ontology functional enrichment analysis of the differentially expressed proteins in this group showed that the most significant cellular component term corresponds to extracellular exosomes in agreement with the EVs nature of the immunocaptured material (**Figure 6D**). Biological process terms of proteins upregulated after 8 months showed that the most significant biological process terms were platelet degranulation and mitochondrial electron transport (**Figure 6E**).

## Discussion

In this study, we present a proteomic analysis of circulating extracellular vesicles (EVs) in COVID-19 patients, examining their clinical progression over two time points separated by eight months. Our clinical data, along with assessments of humoral immune responses—including the detection of IgG, IgA, and IgM antibodies against the S and N proteins of SARS-CoV-2 and their corresponding neutralizing activity align with previously published results for both severe and mild/asymptomatic COVID-19 cases. One of the strengths of this study is the utilization of three distinct and complementary EV isolation methods: CD9^+^ affinity immunocapture, mS1 EV-ganglioside capture, and size-exclusion chromatography (SEC) for proteomic analysis. Importantly, across all tested strategies, no viral peptides associated with EVs were detectable in plasma-derived EVs from COVID-19 patients with varying clinical outcomes. Additional proteomic analysis conducted on an *in vivo* model using SARS-CoV-2-infected hamsters confirmed the same result. This control, not included in previous studies, adds to the robustness of our observations and conclusions. These findings suggest, as previously reported (Ghosh et al., 2020), that the egress of SARS-CoV-2 is largely independent of EV biogenesis.

The most frequently reported symptoms in our patient series were fever, cough, and shortness of breath, aligning with recent meta-analytical data where fever and cough were identified as the most prevalent symptoms associated with severe COVID-19 (Meng et al., 2021). Asthenia was also widely reported (Huang et al., 2020). Notably, 93% of individuals in G1 had pulmonary infiltrates on chest X-rays suggestive of COVID-19 (Shakaib et al., 2021). Regarding comorbidities, hypertension was the most prevalent in G1, consistent with previous reports. DM and previous cardiovascular diseases were also more common among G1 individuals compared to G2 and controls (Shakaib et al., 2021). Among all the laboratory markers, levels of CRP, LDH, procalcitonin, ferritin, hypochromic RBCs, total protein, albumin, calcium, and D-dimer showed the strongest correlation with disease severity. Most of these markers have been recognized as prognostic biomarkers for SARS-CoV-2 (Shakaib et al., 2021; Varikasuvu et al., 2021). However, hypochromic RBCs, total protein, and calcium have not been reported as prognostic markers in other studies, warranting further investigation.

To assess the impact of infection on the immune system, we profiled the S- and N-specific Ig subclasses and their corresponding neutralizing abilities during acute infection and, when available, eight months later. Antibodies were similarly present in most cases during acute infection, with undetectable cases likely representing very early infections (Trinité et al., 2021). The presence of specific antibodies was maintained or increased eight months later due to infection progression, the first vaccine dose, or new infection events, indicating the activation of a humoral adaptive immune response and suggesting that systemic blood-associated protein changes might be occurring in these individuals (Haveri et al., 2021; Pradenas et al., 2021; Trinité et al., 2021).

We report a new and innovative EVs purification methodology based on the interaction of Siglec-1/CD169 with gangliosides on the EVs membrane surface. We developed a bead-based system containing the sialic acid-binding domain of Siglec-1, which strongly binds to surface-exposed gangliosides (Perez-Zsolt et al., 2019). This tool was used as a complementary approach for the proteomic analysis of circulating EVs in COVID-19 patients. Our results showed that EVs captured by mS1 beads from severe patients are enriched in EV markers and contain similar molecular signatures of severity as those captured by CD9^+^ immunocapture, suggesting comparable EV capture potential.

Our initial hypothesis was supported on previous reports indicating that during viral infections, EVs contained viral peptides (Montaner-Tarbes et al., 2016), contribute to immune response amplification, and in specific cases, have been therapeutically applied in immunization strategies (Montaner-Tarbes et al., 2018; Sabanovic et al., 2021; Buzas, 2023; Kalluri, 2024). Our results suggest that this may not be the case for SARS-CoV-2. However, the failure to confirm the primary hypothesis does not invalidate the potential of exploiting EVs containing viral peptides for vaccine design in other viral infections. This concept remains particularly relevant in cases where EVs and viruses utilize the same biosynthetic secretory pathway to exit infected cells.

Beyond this hypothesis, the sets of EV-associated human peptides identified during different stages of the infection provided additional, valuable descriptive proteomic information. Interestingly, our differential expression analysis of CD9^+^EVs cargo from severe COVID-19 patients and non-COVID-19 individuals showed two distinctive signatures of anti-viral response (**Figure 6C**). Specifically, Galectin-3 binding protein, a cell adhesion surface protein with reported antiviral activity and considered among the most conserved exosomal markers (Kugeratski et al., 2021), was among the most abundant proteins in severe patients, as previously reported (Geyer et al., 2021). A recent study found that this protein can bind SARS-CoV-2 S glycoprotein and, when overexpressed, inhibits S-pseudoparticle uptake and spike-induced cell-cell fusion *in vitro* (Gutmann et al., 2021). Additionally, EVs from severe patients isolated by CD9^+^ immunocapture and mS1-EVs trapping showed increased amounts of IFITM3, an antiviral response protein previously associated with EVs in other viral and bacterial infections (Yi et al., 2021; Zou et al., 2021). To our knowledge, this protein has not been previously identified in EVs from COVID-19 patients.

One important observation from our study emerges from the analysis of CD9^+^ EVs in patients eight months post-COVID-19 infection. Severe patients re-evaluated after eight months showed a slight decreased in the total number of EVs proteins. Unexpectedly, the statistical comparison of protein abundance revealed no significant differences, except for a few proteins that showed up- and down-regulation. This suggests that EVs retain a cargo signature bearing traces of protein cargo from peak infection that do not diminish with remission, potentially indicating ongoing pathology. The lack of clinical data from severe patients eight months post-infections limits our ability to correlate this finding with the patients’ health status, preventing confirmation of this hypothesis. Further research is necessary to explore the potential use of EVs as markers for poor convalescence or post-COVID-19 condition.

Interestingly, the most striking result comes from the comparison of uninfected healthcare workers at the initial time point and eight months after. The protein cargo abundance of CD9^+^ EVs showed 124 up-regulated and 70 down-regulated proteins, indicating a substantial change independent of COVID-19 infection. We attribute this change to the single dose of the COVID-19 vaccine received by 70% of individuals in this cohort at eight months post-sample collection. This observation suggests that the vaccine-induced immune response results in a significant reconfiguration of circulating EVs, a hallmark that could be interesting to explore as a vaccine response marker in the future.

Several other proteomics studies also failed to detect viral peptides associated with EVs in COVID-19 patients (Song et al., 2020; Wendt et al., 2020; Fujita et al., 2021; Lam et al., 2021; Mao et al., 2021; Sur et al., 2021; Kawasaki et al., 2022; Moraes et al., 2022). Nonetheless, all these studies, have also identified human molecular signatures. Notably, these signatures have demonstrated their association with disease severity, differentiating between mild and severe cases (Wendt et al., 2020; Fujita et al., 2021), inciting proinflammatory responses in remote cells (Sur et al., 2021), and forecasting the activation of the complement system and heightened platelet reactivity within procoagulant EVs derived from COVID-19 patients (Moraes et al., 2022). Furthermore, these investigations have unveiled a substantial increase in sterols during symptomatic periods (Lam et al., 2021), lipid profiles closely resembling the composition of exosomal membranes (Song et al., 2020), and a lipid metabolic response to oxidative stress-induced oxygen-containing compounds (Mao et al., 2021). Altogether, these data reinforce the importance of EVs in the context of COVID-19 progression and their potential as biomarkers of different clinical manifestations.

One limitation of this study is the inability to conduct a systematic MS analysis using all three different methodologies employed to isolate circulating EVs from COVID-19 patients within the same individuals across the three groups. This constraint arises from the limited availability of plasma samples. However, when feasible, the results demonstrated that the primary findings from the individual MS analyses of the various technologies–such as the total number of proteins, common and unique proteins, and GO terms–were replicated. Another constraint in the study pertains to the system used to generate the recombinant truncated version of mS1, which might contain residual EVs binding to mS1 after its secretion in the culture supernatant of transgenic HEK293T cells. Efforts are currently underway to enhance this system, addressing this limitation and creating a tool that can be widely implemented in future EVs studies.

In summary, we conducted a comprehensive proteomic analysis of circulating extracellular vesicles (EVs) in COVID-19 patients at two longitudinal time points, separated an eight-month interval. Our clinical data largely corroborate the findings of a recent meta-analysis on clinical features and laboratory parameters in COVID-19 patients (Meng et al., 2021), validating the robustness of our patient stratification based on symptomatology and prognostics. To characterize the protein cargo associated with EVs in COVID-19 patients, we employed three distinct EV isolation methods: CD9^+^ affinity immunocapture, mS1 EV-ganglioside capture, and size-exclusion chromatography. Our analyses did not detect any viral peptides in plasma-derived EVs from COVID-19 patients, a finding consistent with other studies and our *in vivo* experimental infection model using Syrian hamsters. The study also revealed specific human molecular signatures within EVs that may serve as potential predictors of disease severity and immune responses. Notably, we observed that vaccine-induced immune response in uninfected healthcare workers led to a significant reconfiguration of circulating EV profiles, a hallmark that could be interesting to explore as a vaccine response biomarker in future studies.

## Data Availability

The datasets presented in this study can be found in online repositories. The names of the repository/repositories and accession number(s) can be found in the article/**Supplementary Material**. These data are already publically available: https://www.ebi.ac.uk/pride/archive/projects/PXD041931
