# Supplementary figures and images for: Proteomics of circulating extracellular vesicles reveals diverse clinical presentations of COVID-19 but fails to identify viral peptides
Source: Front Cell Infect Microbiol. 2024 Nov 6;14:1442743. doi: 10.3389/fcimb.2024.1442743 (PMC11576438; doi:10.3389/fcimb.2024.1442743)

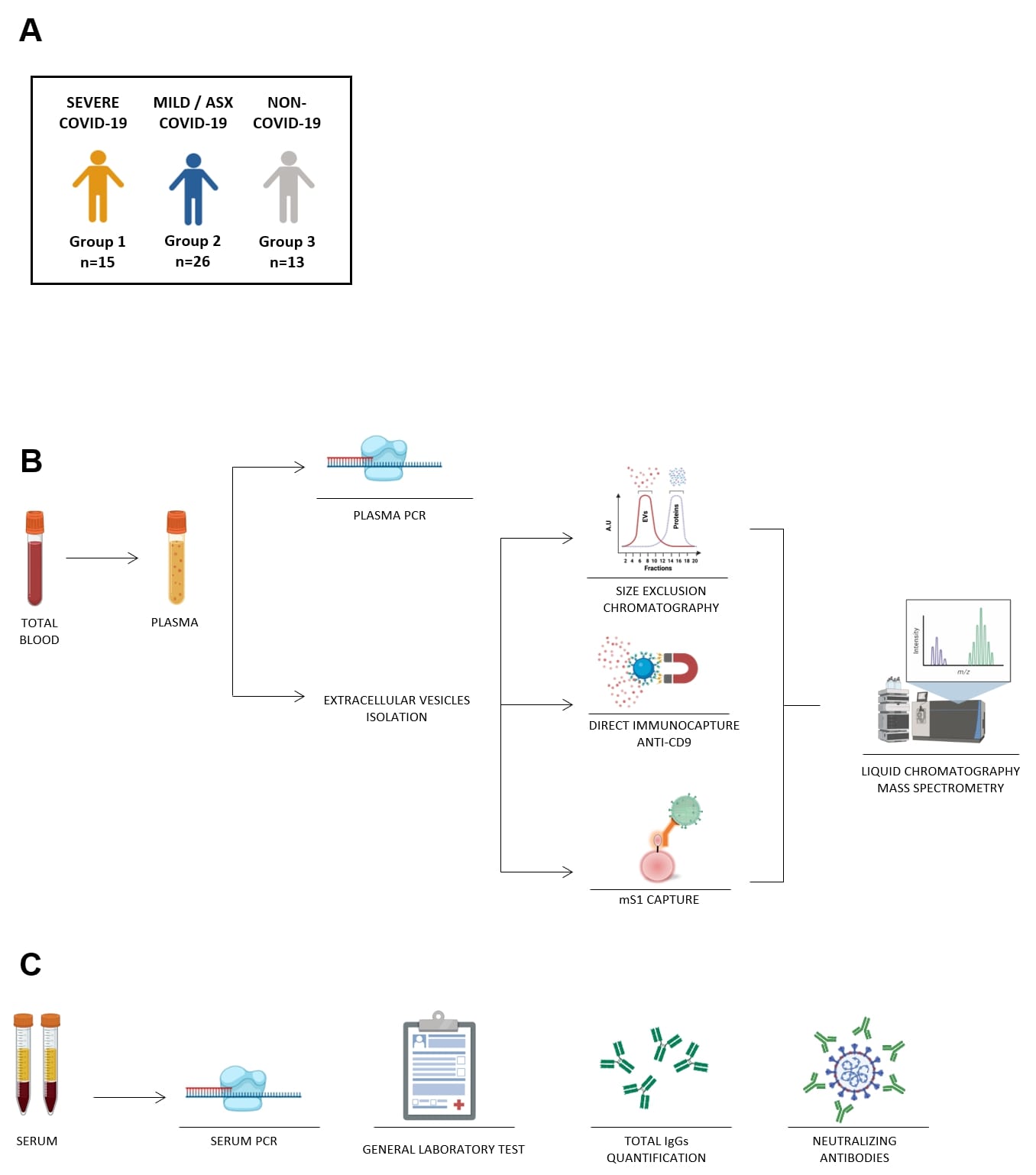

Supplement: Supplementary file 1 [file Image1.jpeg]

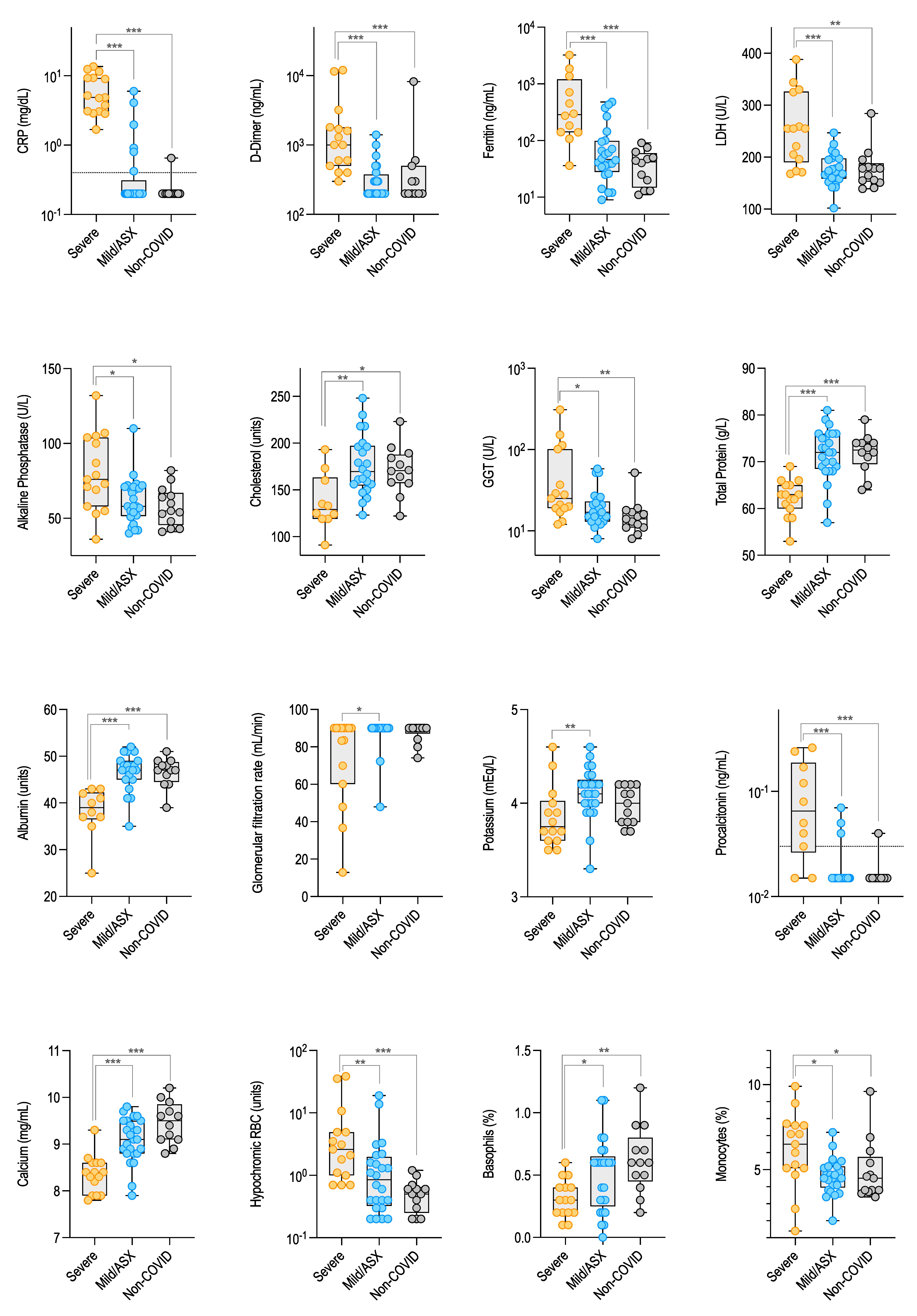

Supplement: Supplementary file 2 [file Image2.jpeg]

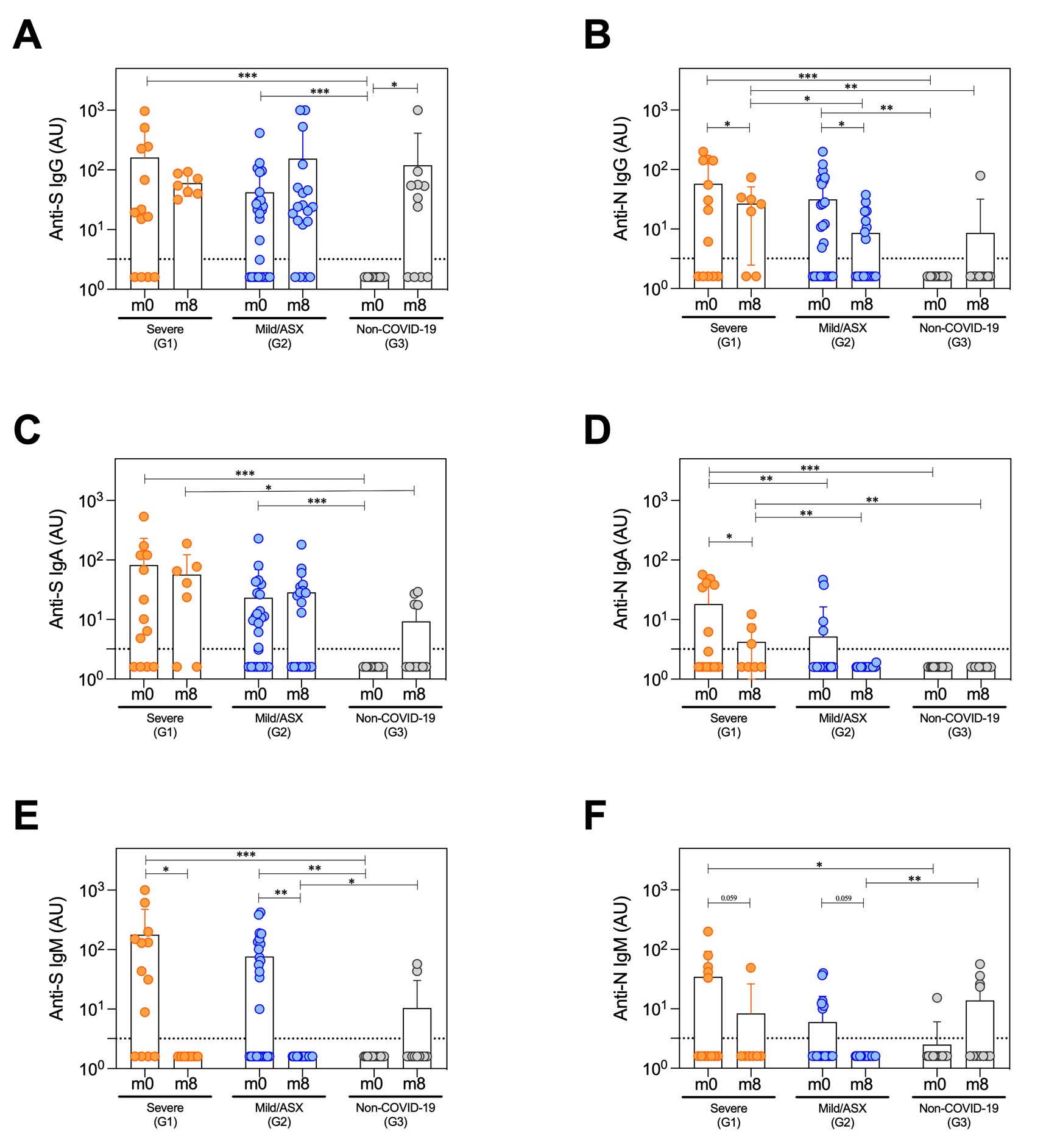

Supplement: Supplementary file 3 [file Image3.jpeg]

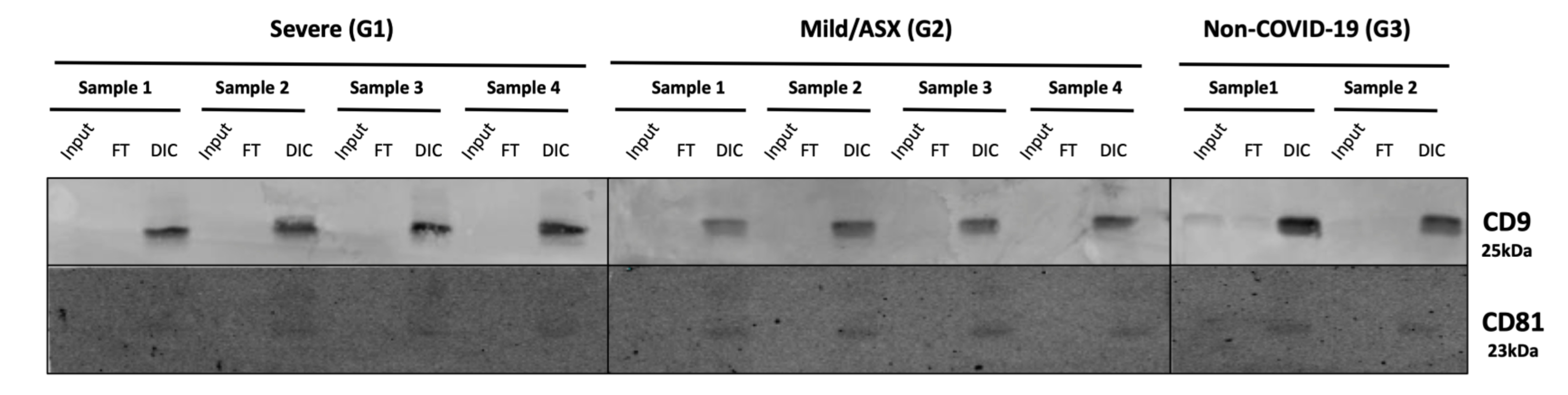

Supplement: Supplementary file 4 [file Image4.jpeg]

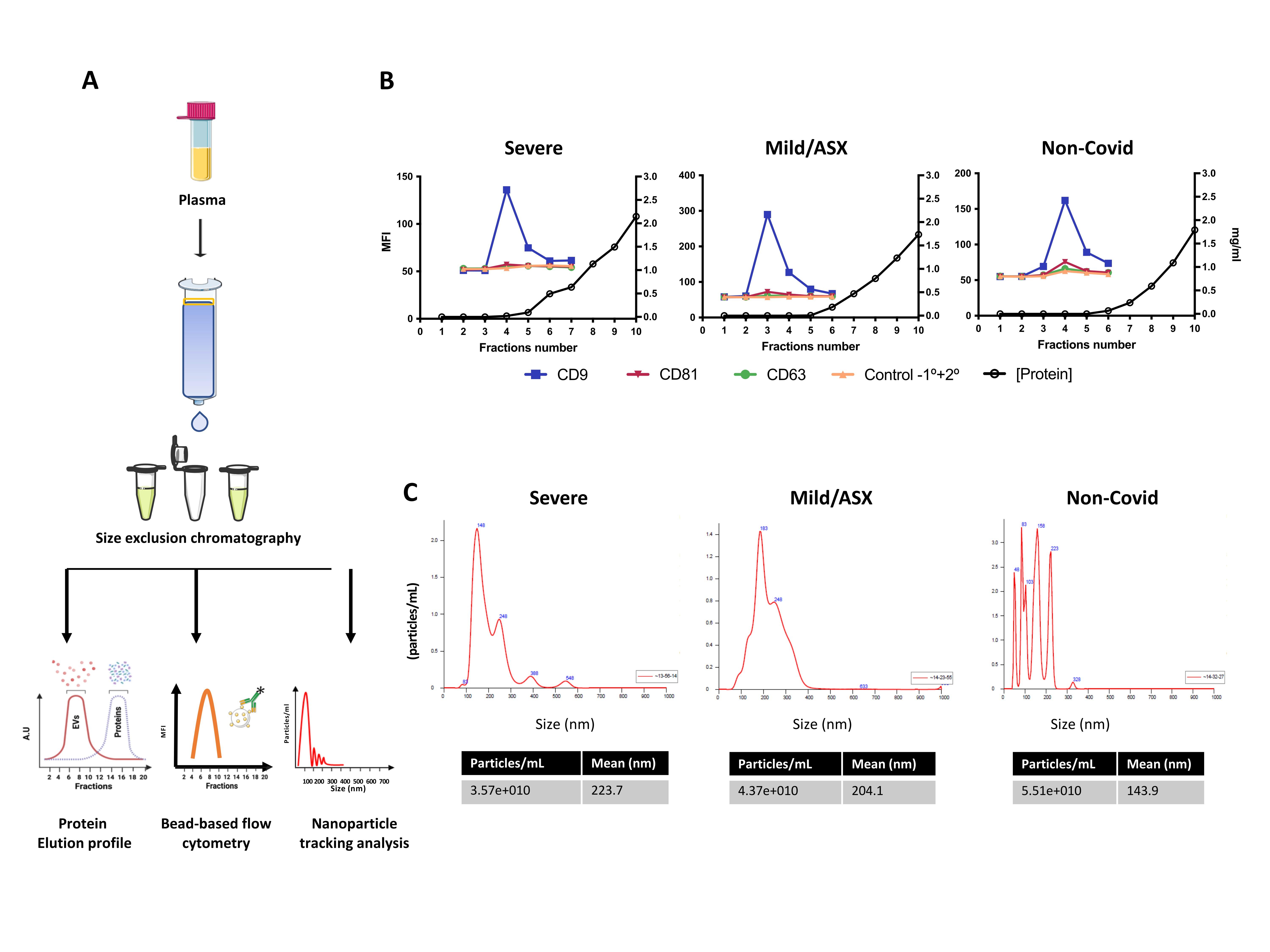

Supplement: Supplementary file 5 [file Image5.jpeg]

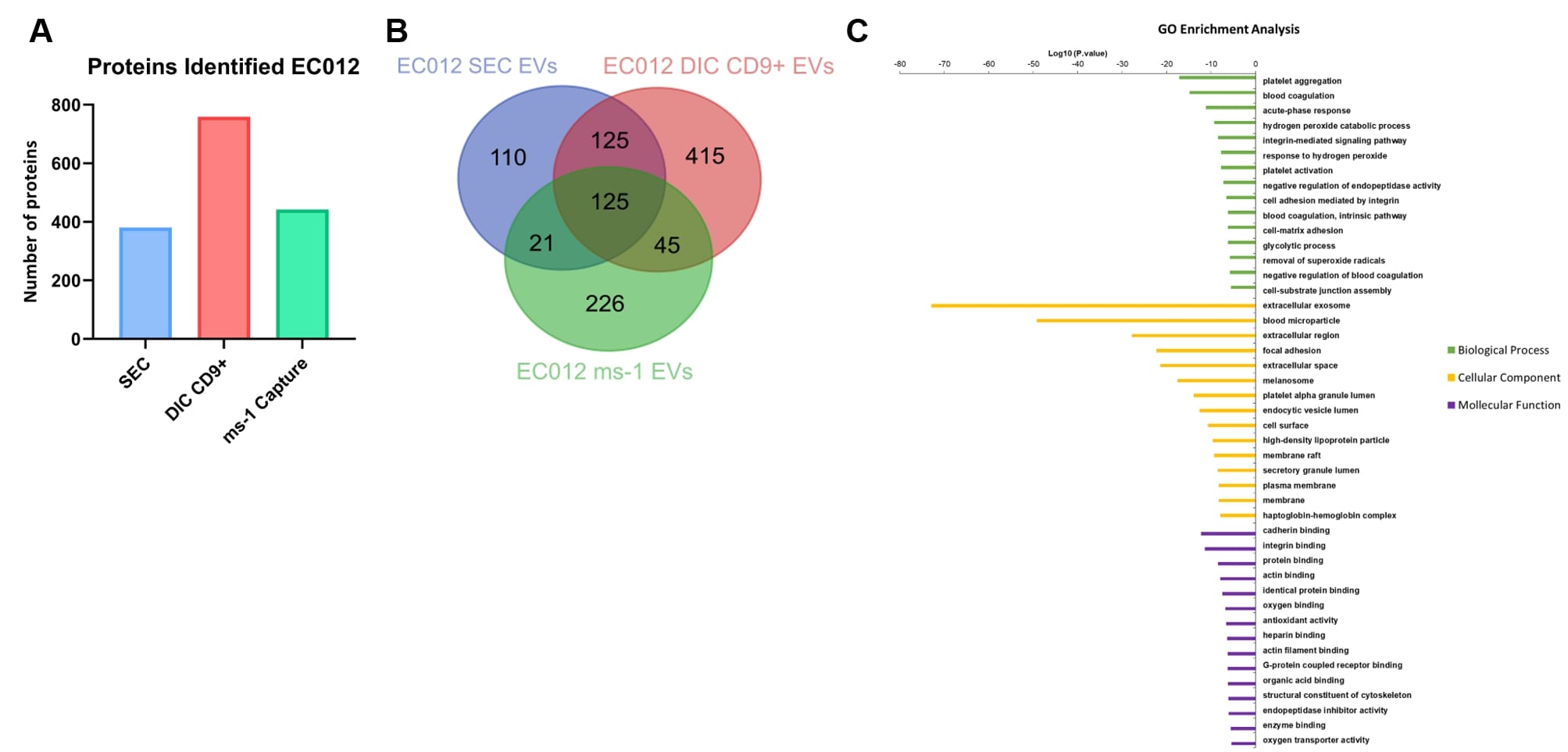

Supplement: Supplementary file 6 [file Image6.jpeg]

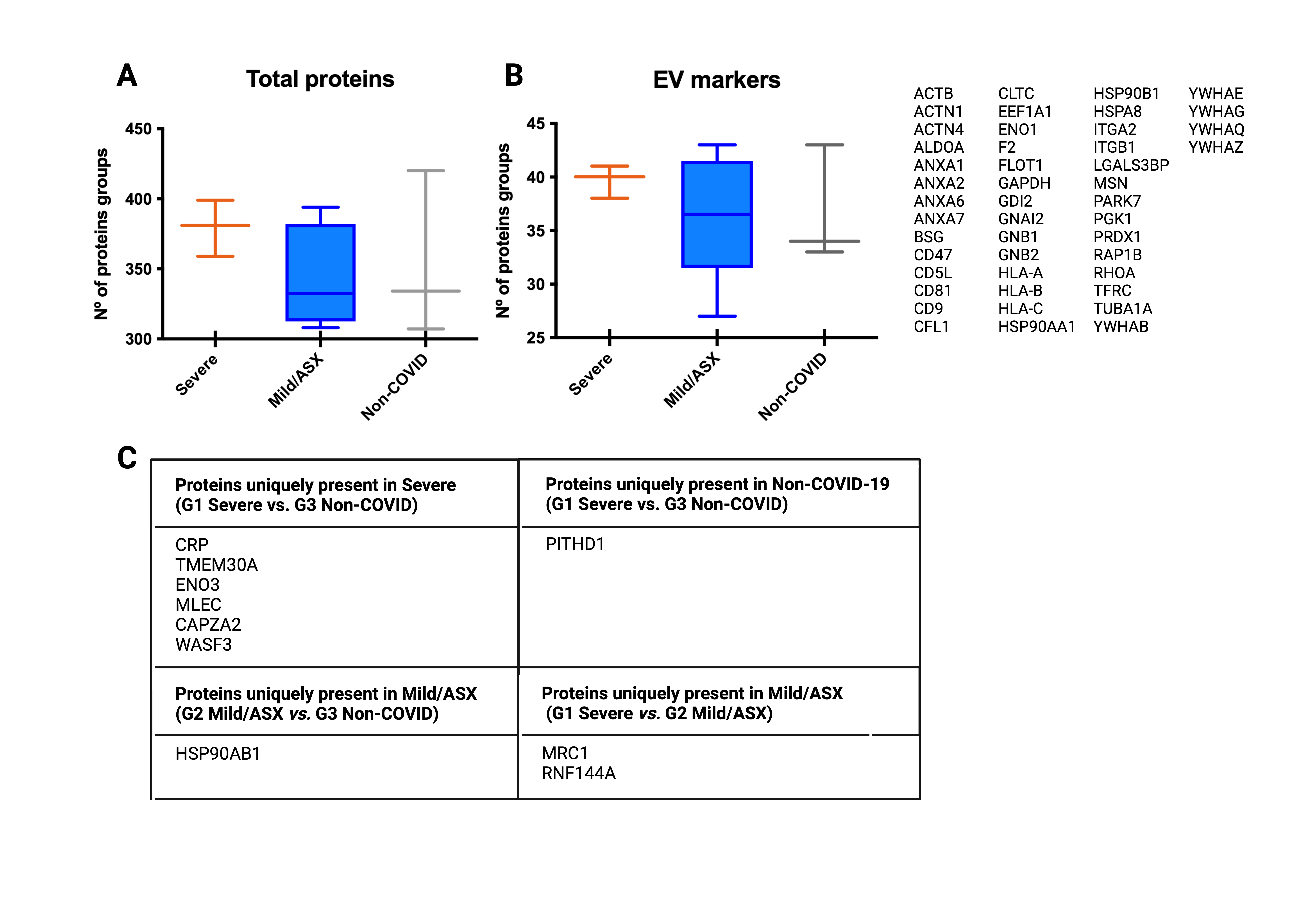

Supplement: Supplementary file 8 [file Image8.jpeg]

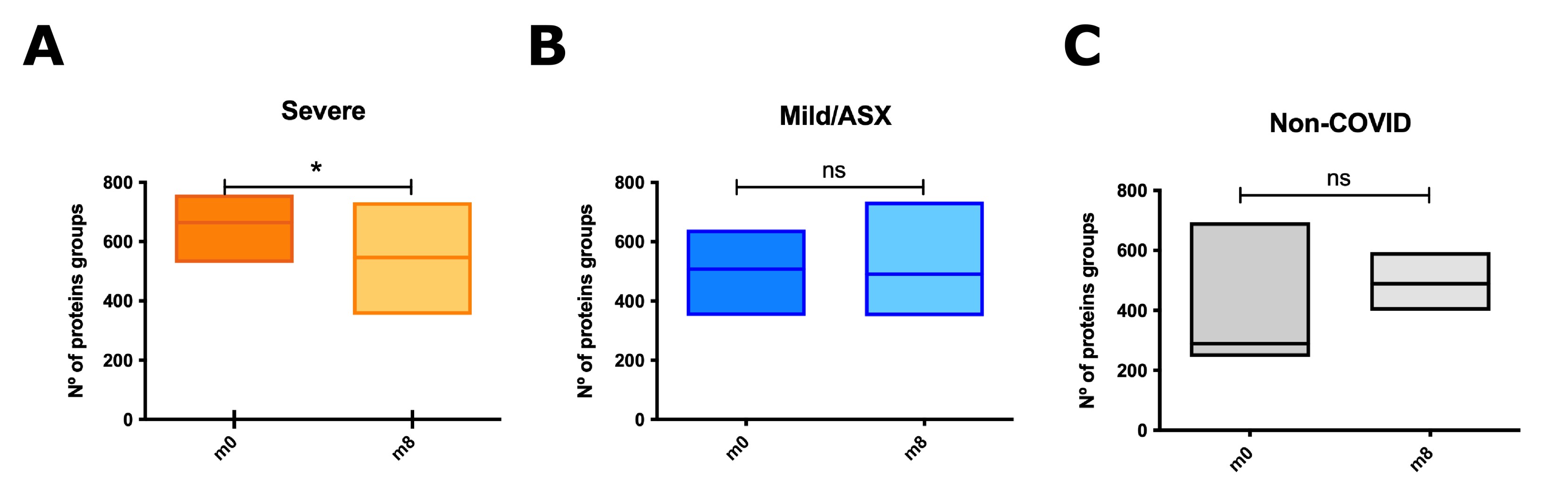

Supplement: Supplementary file 9 [file Image9.jpeg]
